# Physiological mechanisms contributing to the QTL-combination effects on improved performance of IR64 rice NILs under drought

**DOI:** 10.1093/jxb/eru506

**Published:** 2015-02-13

**Authors:** Amelia Henry, B. P. Mallikarjuna Swamy, Shalabh Dixit, Rolando D. Torres, Tristram C. Batoto, Mervin Manalili, M. S. Anantha, N. P. Mandal, Arvind Kumar

**Affiliations:** ^1^International Rice Research Institute, Los Baños, Laguna 4031, Philippines; ^2^Central Rainfed Upland Rice Research Station, Hazaribag, Jharkand 825 301, India

**Keywords:** Canopy temperature, drought, near-isogenic lines (NILs), quantitative trait loci (QTLs), rice, root hydraulic conductivity, roots.

## Abstract

Characterizing the physiological mechanisms behind major-effect drought-yield quantitative trait loci (QTLs) can provide an understanding of the function of the QTLs—as well as plant responses to drought in general. In this study, we characterized rice (*Oryza sativa* L.) genotypes with QTLs derived from drought-tolerant traditional variety AdaySel that were introgressed into drought-susceptible high-yielding variety IR64, one of the most popular megavarieties in South Asian rainfed lowland systems. Of the different combinations of the four QTLs evaluated, genotypes with two QTLs (*qDTY*_*2.2*_ + *qDTY*_*4.1*_) showed the greatest degree of improvement under drought compared with IR64 in terms of yield, canopy temperature, and normalized difference vegetation index (NDVI). Furthermore, *qDTY*_*2.2*_ and *qDTY*_*4.1*_ showed a potential for complementarity in that they were each most effective under different severities of drought stress. Multiple drought-response mechanisms were observed to be conferred in the genotypes with the two-QTL combination: higher root hydraulic conductivity and in some cases greater root growth at depth. As evidenced by multiple leaf water status and plant growth indicators, these traits affected transpiration but not transpiration efficiency or harvest index. The results from this study highlight the complex interactions among major-effect drought-yield QTLs and the drought-response traits they confer, and the need to evaluate the optimal combinations of QTLs that complement each other when present in a common genetic background.

## Highlight

Drought-yield QTLs *qDTY*_*2.2*_ and *qDTY*_*4.1*_ improved rice variety IR64 complementarily through peak QTL effects at distinct stress levels, and by increasing root hydraulic conductance and root growth at depth.

## Introduction

Several major-effect drought-yield quantitative trait loci (QTLs) have been reported in rice: *qDTY*_*12.1*_ (Bernier *et al.*, 2007), *qDTY*_*3.1*_ (Venuprasad *et al.*, 2009), *qDTY*_*1.1*_ (Vikram *et al.*, 2011), and four QTLs from an IR64 × AdaySel cross (Swamy *et al.*, 2013). These QTLs were detected by genetic mapping for yield under drought; as such, no physiological drought response mechanisms were selected for or characterized during the development of breeding lines with those QTLs. This top-down approach has allowed the focus of selection to be on grain yield in intact crop plants, rather than extrapolation of component traits or genes (Sinclair, 2011). Besides providing an understanding of the functional significance of a particular genetic region, phenotyping of the mechanisms/traits associated with a QTL has the potential to further contribute to the breeding process in developing selection tools (Passioura, 2012). Characterizing the drought-yield QTLs that have been detected in rice could facilitate their selective combination in future drought breeding work. Identifying the mechanistic drought responses of drought-tolerant lines could also help define the most appropriate target environments for these improved genotypes, by better understanding their response to specific drought conditions. Furthermore, working with genetic material that is known to have improved yield under drought, and being able to compare highly genetically similar germplasm that results in contrasting yield under drought [i.e. drought QTL near-isogenic lines (NILs) and the recipient parent or –QTL lines], may lead to the identification of novel drought-resistance traits.

A cross of the improved but drought-susceptible rice variety IR64 and the traditional, stress-tolerant variety AdaySel resulted in the identification of four AdaySel-derived major-effect drought-yield QTLs in backcross-inbred lines (BILs; Venuprasad *et al.*, 2007). Subsequently, NILs were developed with two QTLs that showed improved yield (0.5–1.9 t ha^–1^) under drought in farmers’ fields in South Asia and similar yield to the recurrent parent (IR64) under well-watered conditions (Swamy *et al.*, 2013). Transcript profiling of the 4-QTL BILs suggested that different physiological mechanisms were associated with drought resistance in different BILs (Moumeni *et al.*, 2011), and an analysis of differentially expressed genes within the four QTL regions pointed to root development and function as contributing to drought resistance in the BILs (Swamy *et al.*, 2013). On a whole-plant scale, the +QTL BILs showed large differences in transpiration under severe stress (as evidenced by stomatal conductance and canopy temperature) compared to –QTL BILs and IR64 (Venuprasad *et al.*, 2011; Swamy *et al.*, 2013). Despite the significant effect of the four QTLs on yield and transpiration under drought, as well as strong support in the literature for root growth at depth to confer drought resistance in rice [as reviewed by Gowda *et al.* (2011) and Comas *et al.* (2013)], the BILs did not show greater root length at depth under drought in three field experiments (Swamy *et al.*, 2013). This unexpected result highlighted the need for more detailed physiological characterization to explain the improved yield under drought seen in the lines derived from the IR64 × AdaySel cross.

The objectives of this study were to characterize the drought-response mechanisms of AdaySel-derived QTL lines in the IR64 background. Genetic material included BILs and subsequently NILs with different QTL combinations that were being developed concurrently to this physiological characterization. The large effect on yield and the genetic characterization of the genotypes studied also allowed for a further evaluation of the interactions among multiple drought-yield QTLs and their effects on drought responses in field conditions.

## Materials and methods

### Genetic material

There were three components to this study, each of which used different genetic material: (i) evaluation of the effects of drought-yield QTLs *qDTY*_*2.2*_ and *qDTY*_*4.1*_ across a range of drought-stress severities; (ii) evaluation of QTL combinations; and (iii) detailed physiological characterization. All studies used BILs and NILs of rice (*Oryza sativa* L.) derived from the cross of IR64 and AdaySel, which was the donor of *qDTY*_*2.2*_, *qDTY*_*4.1*_, *qDTY*_*9.1*_, and *qDTY*_*10.1*_ (Table 1). For (i) the evaluation of *qDTY*_*2.2*_ and *qDTY*_*4.1*_ effects across a range of stress severities, available data was used from Dixit *et al.* (2012) and Swamy *et al.* (2013) on the IR77298-14-1-2 × IR64 population from seven experiments conducted under drought stress and well-watered conditions. For (ii) the evaluation of QTL combinations, NILs were developed with either IR77298-14-1-2-10 or IR77298-5-6-18 as the donor parent. Among lines with different combinations of the four QTLs, the best NILs for yield under drought were IR87707-445-B-B-B and IR87707-446-B-B-B, NILs that were 97% genetically similar to IR64 with two QTLs (*qDTY*_*2.2*_ and *qDTY*_*4.1*_), and IR77298-14-1-2-10 as the donor parent (Swamy *et al.*, 2013); these were used for (iii) detailed physiological characterization. The detailed physiological characterization also included BILs IR77298-5-6-18 and IR77298-14-1-2-10 that were classified as ‘+QTL’ based on detection of QTLs under severe drought stress, and IR77298-5-6-11 and IR77298-14-1-2-13 that were classified as ‘–QTL’ based on no detection of QTLs under severe drought stress. Subsequent evaluation under mild and moderate stress revealed the presence of *qDTY*_*4.1*_, but since the focus of the physiological characterization in this study was severe drought stress, the term ‘–QTL’ was used here.

**Table 1.** Genetic identity of the IR64 × AdaySel-derived BILs and NILs used in this study for detailed physiological characterization

Genotype	Generation	QTL from AdaySel				Genetic similarity to IR64 (%)
		*qDTY*_*2.2*_	*qDTY*_*4.1*_	*qDTY*_*9.1*_	*qDTY*_*10.1*_	
**QTL-combination studies**						
IR64						
IR87705-72-12-B	NIL	√				96.5
IR87705-83-12-B	NIL	√			√	95.0
IR87705-85-4-B	NIL	√				94.7
IR87705-6-8-B	NIL		√			95.5
IR87705-7-15-B	NIL		√			93.9
IR87705-80-15-B	NIL		√		√	94.6
IR87705-25-4-B	NIL		√			94.4
IR87705-44-4-B	NIL		√		√	96.2
IR87705-14-11-B	NIL	√	√		√	96.7
IR87707-118-B-B-B	NIL	√	√			95.8
IR87707-186-B-B-B	NIL	√	√		√	96.9
IR87707-445-B-B-B	NIL	√	√			96.9
IR87707-446-B-B-B	NIL	√	√			97.0
IR87728-491-B-B	NIL	√	√	√		92.6
IR87729-69-B-B-B	NIL	√	√	√	√	94.4
**Physiology studies**						
IR64						
AdaySel		√	√	√	√	
IR77298-14-1-2-B-10 (+)	BIL/ BC_3_F_3_ derived	√	√			-
IR77298-14-1-2-B-13 (–)	BIL/ BC_3_F_3_ derived		√^b^			-
IR77298-5-6-B-18 (+)	BIL/ BC_3_F_3_ derived		√			-
IR77298-5-6-B-11(–)	BIL/ BC_3_F_3_ derived		√^b^			-
IR87707-445-B-B-B	NIL	√	√			96.9
IR87707-446-B-B-B	NIL	√	√			97.0
IR87707-182-B-B-B	NIL	√	√			96.9
IR87729-69-B-B-B	NIL	√	√	√	√	94.4

^a^ Development of genotypes is described by Swamy *et al.* (2013).

^b^ Detected only under moderate drought stress, and not under severe drought stress.

### Evaluation of *qDTY*_*2.2*_ and *qDTY*_*4.1*_ effects across a range of drought stress severities

Experiments to evaluate the effects of *qDTY*_*2.2*_ and *qDTY*_*4.1*_ under varying severities of drought stress were conducted under lowland transplanted conditions in replicated trials with an alpha lattice design during the 2007 dry season (DS), 2007 wet season (WS), and 2008DS at the International Rice Research Institute (IRRI, Los Baños, The Philippines, 14°10′11.81″N, 121°15′39.22″E). Phenotypic and genotypic data [as described by Dixit *et al.* (2012), and Swamy *et al.* (2013)] of 288 lines from the 2007WS and 2008DS were used for the analysis, and subsets of 154 and 134 lines were used from the two 2007DS experiments, respectively, to conduct the analysis. The difference between mean yield values of lines with and without QTLs based on peak markers RM555 for *qDTY*_*2.2*_ and RM518 for *qDTY*_*4.1*_ were calculated and used to obtain the percentage advantage due to the presence or absence of the AdaySel allele at these loci under different severities of stress. The percent yield advantage data for *qDTY*_*2.2*_ and *qDTY*_*4.1*_ were plotted against population means of the respective experiments, the data were fitted with non-linear regression curves using SigmaPlot 11.0 (Systat Software, Inc.), and the two curves were compared by the general linear model in SAS version 9.3 (SAS Institute).

### Field reproductive stage drought physiology studies

Six separate lowland field studies were conducted in rainout shelters to evaluate the QTL combinations and to characterize the physiological response of BILs and/or NILs with different AdaySel-derived QTLs; there were five at IRRI and one at the Central Rainfed Upland Rice Research Station (CRURRS; Hazaribag, India, 23°57′39.30″N, 85°22′5.07″E). Experiments were conducted during the 2011WS (June 2011–November 2011), 2012DS (December 2011–April 2012), 2012WS (CRURRS; June 2012–November 2012), 2013DS (December 2012–April 2013), and 2013WS (June 2013–November 2013). The experiments at CRURRS were part of the AICRIP (All India Coordinated Rice Improvement Programme) for evaluation before varietal release. Soil particle size distribution was 38% clay, 21% sand, and 41% silt at IRRI; and 34% clay, 30% sand, and 36% silt at CRURRS. Bulk density at 30cm averaged 1.13g cm^–3^ at IRRI and 1.69g cm^–3^ at CRURRS (see Supplementary Tables S1 and S2 for other soil and ambient conditions). All trials were managed according to the protocol described by Henry *et al.* (2011), including transplanting, hand-weeding, and snail control. No pest control was necessary at CRURRS. In each season, seedlings were transplanted at 3 weeks after sowing into puddled soil, with 0.2 m between hills and 0.25 m between rows of 3 m in length (4 rows per plot). Four replicates per genotype were planted in each trial (except in 2013DS when three replicates were planted) and arranged in randomized compete block designs. Basal fertilizer was applied as basal at the rate of 40-40-40 (N-P_2_O_5_-K_2_O) kg ha^–1^ at the time of transplanting, with one topdressing of 40kg N ha^–1^ using (NH_4_)_2_SO_4_ applied at 3 weeks after transplanting [about 42 days after sowing (DAS), or at least 4 days before draining of the field to impose drought stress]. The basal fertilizer application was omitted in the 2013DS experiment to test response to low-fertility conditions. Fields were drained 4 weeks after transplanting (49 DAS) to impose drought stress. Rainfall was excluded from the experimental field using an automated rolling rainout shelter at IRRI and a stationary rainout shelter at CRURRS. Plants in the drought-stress treatments were re-watered by flooding, then immediately drained, at 89 DAS in 2012DS, 72 and 112 DAS in 2012WS, 80 and 97 DAS in 2013DS, and 75 and 100 DAS in 2013WS.

#### Measurements in the drought-stress treatment 

Soil water potential was monitored three times per week in all seasons with 2–3 tensiometers installed at a depth of 30cm in each experimental field (Soilmoisture Equipment Corp, CA, USA; Supplementary Figure S1). Diurnal volumetric soil moisture levels were monitored at 20cm and 40cm in each season at IRRI (1–2 sensors per depth per plot, HOBOnode, Onset Computer Corp., Bourne, MA, USA), and also at 10-cm depth increments in 2013WS (Diviner 2000, Sentek Sensor Technologies, Stepney, SA, Australia). Canopy temperature was measured at IRRI with a combination of thermography (NEC TH7800 infrared camera, NEC Avio Infrared Technologies Co. Ltd., Tokyo, Japan, in the 2012DS; and FLIR Thermocam A320 Infrared Camera, FLIR Systems Co., Boston, MA, USA, in the 2013DS) and infrared (IR) sensor (three locations per plot; Apogee Instruments, Logan, UT, USA); and at CRURRS (Agri-Therm III, Everest Interscience Inc., Tucson, AZ, USA). Normalized Difference Vegetation Index (NDVI; Greenseeker Hand-held Sensor, NTech Industries, CA, USA) was measured around midday in the drought-stress treatments throughout all seasons at IRRI.

#### Measurements in the drought-stress and well-watered control treatments 

Xylem sap bleeding rate was determined at IRRI and CRURRS according to Morita and Abe (2002), where shoots of three hills per plot were cut about 10cm above the soil surface, and sap exuded from the cut stems was collected with a pre-weighed cotton towel wrapped in plastic for 4 hours, from about 7–11am. Sap bleeding rate was normalized by shoot mass for each hill. At IRRI, leaf water potential (LWP) was determined as the pressure necessary to force sap from the cut end of the leaf (3000HGBL Plant Water Status Console, Soilmoisture Equipment Corp., CA, USA) across the day in three leaves per plot during the 2012DS (88 DAS) using compressed N_2_. Leaf relative water content (LRWC) was calculated at the same time as LWP in 2012DS as

$$\begin{array}{l} \mathrm{LRWC}=\left(\text{drought }-\text{ stressed}\;\text{leaf weight }-\text{ leaf dry weight}\right)\\ \;=\;\left(\text{turgid leaf weight }-\text{ leaf dry weight}\right)\times 100 \end{array}$$

where the turgid leaf weight was determined after saturating the leaf in de-ionized water overnight. In 2012WS, one plant per plot of IR87707-445-B-B-B and IR64 from the field (90–91 DAS) were excavated around the root crown (including soil) into 10-l buckets, root zones were re-watered overnight, and leaves were used for LWP and LRWC measurements the next day after allowing them to dry on a work bench for 2–90 minutes after cutting. Carbon isotope discrimination (Δ^13^C) was calculated as

$$\left(–\text{8}-{\text{leaf}}^{\text{13}}\text{C concentration}\right)/\left[\text{1}+\left({\text{leaf}}^{\text{13}}\text{C concentration}/\text{1}000\right)\right]$$

according to Farquhar *et al.* (1989) from the youngest fully-formed leaves (nine per plot) collected at 79 and 114 DAS in 2012DS and 2013DS. Photosynthesis rates were measured using the LI-6400 portable gas exchange system (Li-Cor Inc., Lincoln, NE, USA). Leaf area (three hills per plot; LI-3100C, Li-Cor Inc., Lincoln, NE, USA) was measured at 79 DAS in 2012DS; 57, 67, 80, 94, and 108 DAS in 2012WS; and 114 DAS in 2013DS. Shoot mass was measured at the time of all leaf area and bleeding rate measurements. Leaf osmotic potential was determined in 2013WS by collecting 3–5 of the youngest fully-expanded leaves per plot, freezing the tissue inside a 5-ml syringe at –15°C, briefly thawing the sample, pressing the sap from the leaf tissue, and measuring 10 µl with a vapour pressure osmometer (Vapro model 5520, Wescor, Logan, UT, USA). For a comparison of different leaf water status parameters, results were standardized to have a mean of 0 and a standard deviation of 1 using the ‘standardize’ transformation in SigmaPlot 11.0 (Systat Software, Inc.).

Root samples were taken at 91 DAS in 2012DS, 73 DAS in 2012WS, 98 DAS in 2013DS, and 102 DAS in 2013WS at IRRI using a 4-cm diameter core sampler (fabricated at IRRI, Los Baños, The Philippines) to a depth of 60cm. Three subreplicate cores per plot were sampled, soil cores were divided into 15-cm segments, and roots were washed by repeatedly mixing the soil with water in a container and pouring the root-water suspension over a 1-mm plastic sieve. Only roots identified as living rice roots were retained for analysis. All samples were stored in 50% ethanol until scanning. Root samples were scanned at 600 dpi (Epson V700, CA, USA), and scanned images were analysed using WinRhizo v. 2007d (Régent Instruments, Québec, Canada). A pixel threshold value of 200 was set for the analysis, and diameter classes of <0.05mm, 0.05–0.1mm, 0.1–0.2mm, 0.2–0.5mm, 0.5–1.0mm, and >1mm were used.

In all field experiments, time to flowering was recorded when 50% of the hills in a plot flowered. Shoot biomass and grain yield were measured after harvesting and threshing the grains. Grain yield values were normalized to a grain moisture content of 14%.

### Greenhouse seedling-stage drought studies

Three seedling-stage greenhouse studies (Lpr1, June–July 2010; Lpr2, January–February 2013; Lpr3, May–June 2013) were conducted to characterize root hydraulic conductivity and anatomy, as well as one additional experiment for root anatomy only (Root anatomy4, September 2013). Soil from the IRRI upland farm (Supplementary Table S1) was dried in a greenhouse, sieved (6mm), steam sterilized, and packed to a bulk density of 1.1 (Lpr1) or 1.2g cm^–3^ (Lpr2, Lpr3, and Root anatomy4) in 5-cm diameter Mylar tubes to a depth of 40cm. All tubes were closed at the bottom with a layer of cotton cloth to allow water flow to the soil, and then inserted inside an outer tube of opaque PVC painted white that had a water-impermeable sealed bottom. Soil moisture treatments of well-watered (WW), dry-down from field capacity (DD; in Lpr1 only), and dry-down from 75% of field capacity (DD-75%) were applied. For the WW and DD treatments, tubes were soaked for 2 hours, then allowed to drain overnight before planting. For the DD-75% treatment, half of the required water volume was added to the top of the tube and half to the bottom, in order to have a continuous water column in that low-moisture treatment. Five replicates per genotype were used for root hydraulic conductivity (*Lp*_*r*_) measurements. Seeds were germinated for 4 days in a 28°C incubator, and then transferred to the soil-filled tubes. Planting was staggered to allow all samples to be 21 days old at the time of *Lp*_*r*_ measurement. Tubes were weighed three times per week to monitor transpiration, at which time WW tubes were watered to maintain 120% of field capacity throughout the experiment. Unplanted controls for each treatment were included to monitor evaporation from the soil surface.

Soil moisture levels at 21 days after planting declined to volumetric water contents of 0.23 and 0.22 in the DD and DD-75% treatments of Lpr1, to 0.19 in the DD-75% treatment of Lpr2, to 0.17 in the DD-75% treatment of Lpr3, and to 0.21 in the DD-75% treatment of Root anatomy4, as affected by the range of ambient conditions among studies (Supplementary Table S2). The *Lp*_*r*_ was measured as described in Henry *et al.* (2012) by cutting the shoots and forcing xylem sap to exude from the cut stem by pressurizing the root zone (within the Mylar tube) in a 1600-cm^3^ pressure chamber (3000HGBL Plant Water Status Console, Soilmoisture Equipment Corp., CA, USA) using compressed air. Each sample was first equilibrated for 10min at 0.2MPa, then xylem sap was collected for 10min at each pressure (0.2, 0.35, and 0.5MPa). In the Lpr1 experiment, plants in the drought treatment were not re-watered before the *Lp*_*r*_ measurement; results integrated both soil and root hydraulic conductivity. In studies Lpr2 and Lpr3, tubes (root zones only) in the DD-75% treatment were soaked in water for about 30min and allowed to drain for 10min prior to the *Lp*_*r*_ measurement in order to reduce the effect of soil conductivity. In Lpr3 an additional treatment of re-watering with 4mM sodium azide was included to compare the genetic responses to aquaporin inhibition.The *Lp*_*r*_ was calculated as the slope of xylem sap flux at increasing pressures when the curve is linear (Matsuo *et al.*, 2009), and normalized for root surface area. After measuring the *Lp*_*r*_, maximum root depth in each tube was determined, and roots were washed and stored in 75% ethanol until scanning and analysis with WinRhizo (Régent Instruments, Quebec, Canada). Leaves from the DD-75% treatment (in which no solution was added) were used for osmotic potential measurements as described above.

### Anatomical observations

From each greenhouse experiment, three nodal roots of each plant were hand-sectioned at 2cm from the root apex, and at the mid-point along the root axis in the Lpr1 experiment. Sections were stained with Sudan IV (Aldrich, USA) according to Zeier *et al.* (1999) to visualize suberin deposits within the root section. Images of five sections per root were acquired at 50× and 100× magnification with a Zeiss Axioplan 2 compound microscope. Section images were analysed for the percentage of the cortex as aerenchyma with GIMP v. 2.5 (GNU Image Manipulation Program; www.gimp.org), and for anatomical attributes including diameter of the root section, stele, and metaxylem vessels using Image J software (Abramoff *et al.*, 2004).

Three of the youngest fully-expanded leaves per plot were collected at 119 DAS (2012DS) and 106 DAS (2012WS) for leaf anatomical observations. Leaves were stored in 70% ethanol until sectioning and imaging at 200× with a Zeiss Axioplan 2 compound microscope. Parameters including large vein and small vein, width, height, leaf thickness, and interveinal distance were determined, as well as large-vein and small-vein xylem diameter and vessel number.

### Statistics

Statistical analyses were performed in R v. 2.15.2 (R Core Team, 2012) to compare genotypes using ANOVA (*aov*) for single parameters in an individual trial with genotype and replication as fixed variables; LSD mean comparison was used as the post-hoc test. For experiments in which repeated measurements were conducted, genotypes were compared with the mixed model ASREML using Wald’s test in R, with ‘QTL combination’ or ‘genotype’ and ‘days after sowing’ as fixed variables and ‘replicate’ as a random variable.

## Results

### Evaluation of *qDTY*_*2.2*_ and q*DTY*_*4.1*_ effects across a range of drought-stress severities

**Fig. 1.**
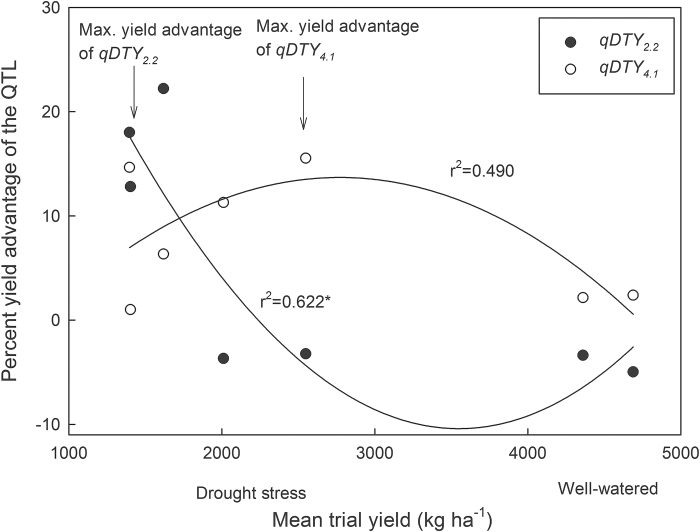
Percent advantage of *qDTY*_*2.2*_ and *qDTY*_*4.1*_ under varying severities of drought stress across seven experiments conducted with the IR77298-14-1-2 × IR64 population. Each point represents the difference between +QTL and –QTL lines in one field study. The total number of lines (+QTL, –QTL, and heterozygotes) ranged from 134 to 288 in each study. The significance of the curve fitting by non-linear regression is indicated: *, *P* < 0.05. The significant interaction between the qDTY category and the quadratic term, *P*-value [(Mean*Mean*qDTY) = 0.036], indicates a difference between the curves.

*qDTY*_*2.2*_ and *qDTY*_*4.1*_ showed distinct patterns of effects on yield under different severities of drought stress (Fig. 1). *qDTY*_*2.2*_ showed a maximum yield advantage in experiments where stress levels were severe and the mean yields were below 1600kg ha^–1^; this yield advantage declined as the stress level decreased (mean trial yield increased). No yield advantage of *qDTY*_*2.2*_ was seen under well-watered conditions. In contrast, *qDTY*_*4.1*_ showed a maximum yield advantage in experiments where stress levels were mild to moderate (mean trial yield of about 3000kg ha^–1^) and declined again under well-watered conditions, although in the case of *qDTY*_*4.1*_ the yield advantage remained positive under well-watered conditions.

### Drought response of lines with different QTL combinations

**Fig. 2.**
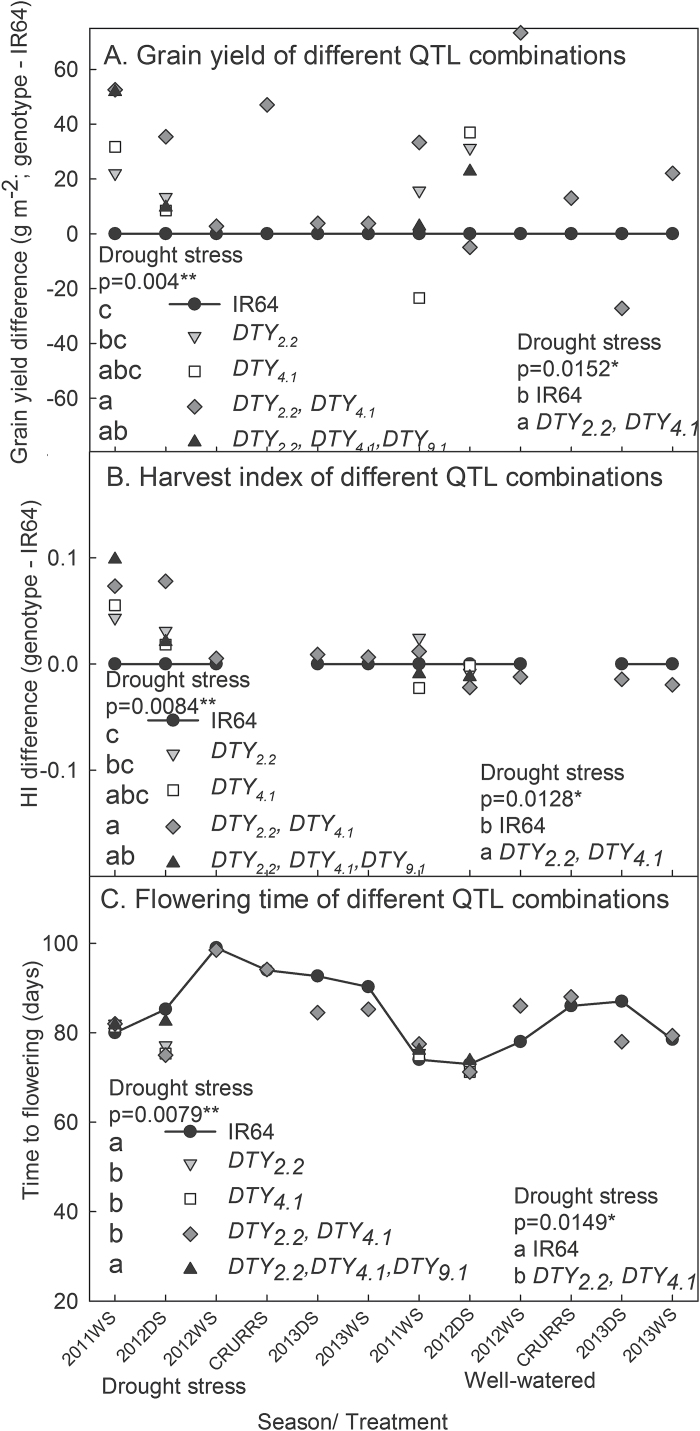
Response of different AdaySel-derived QTL combinations in NILs in the background of IR64. (A) Difference in grain yield of various AdaySel-derived QTL combinations compared to IR64. (B) Difference in harvest index (HI) of various AdaySel-derived QTL combinations compared to IR64. (C) Flowering time of various AdaySel-derived QTL combinations compared to IR64. Each point represents the mean of 2–3 genotypes with that QTL combination. Significant differences shown are for the absolute values, whereas the graphs of yield and HI illustrate the difference from IR64. Results from the statistical analysis of only seasons in which genotypes with all QTL combination were studied are shown on the left, and results from the statistical analysis comparing IR64 and the 2-QTL NILs are shown on the right in each panel. No significant differences across well-watered treatments were observed. The presence of *qDTY*_*10.1*_ is not presented here, and other QTL combination NILs were omitted for simplicity.

**Fig. 3.**
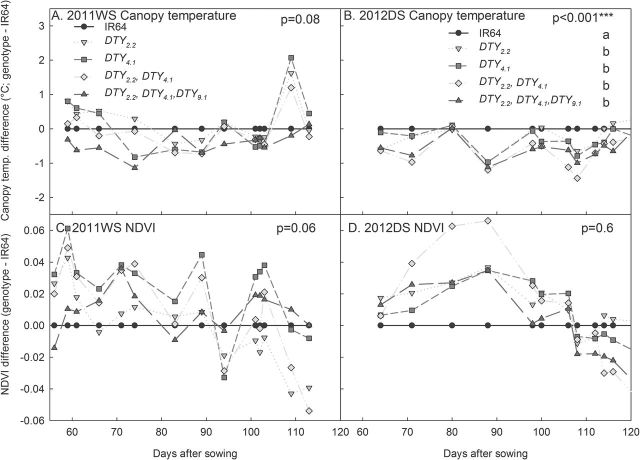
Response of different AdaySel-derived QTL combinations in NILs in the background of IR64. (A, B) Canopy temperature difference from IR64. (C, D) NDVI. Each point represents the mean of 2–3 genotypes with that QTL combination. The presence of *qDTY*_*10.1*_ is not presented here, and other QTL-combination NILs were omitted for simplicity. *P*-values shown are for genotypic differences across the experiment as determined by the mixed model ASREML.

Out of all possible QTL combinations from the four initial QTLs identified from the IR64 × AdaySel cross (Table 1), the 2-QTL NILs (*qDTY*_*2.2*_ + *qDTY*_*4.1*_) showed the significantly highest grain yield and harvest index under severe drought-stress treatments (Fig. 2A–B), but no differences under well-watered conditions were observed. Time to flowering was significantly earliest in lines with *qDTY*_*2.2*_, *qDTY*_*4.1*_, and *qDTY*_*2.2*_ + *qDTY*_*4.1*_ under drought stress, but was similar among lines under well-watered conditions (Fig. 2C). Different levels of drought response were evident in NILs with different QTL combinations in terms of canopy temperature and NDVI, where NILs with combinations of 3–4 QTLs tended to show the least improvement over IR64 (Fig. 3), although this was more evident in the dry season than in the wet season.

### Detailed physiological characterization

#### Above-ground parameters: shoot growth and leaf water status

Shoot biomass was generally larger in all +QTL lines compared with IR64 under drought (Supplementary Figure S2) and in some WW control treatments (Supplementary Figure S3), and these differences were on average greater in the 2-QTL NILs. Leaf area (Supplementary Figure S4) and NDVI under drought (Supplementary Figure S5) showed similar trends to that of shoot biomass. Photosynthesis rates were significantly higher in IR87707-445-B-B-B than IR64 under drought in only one of the three seasons measured (Supplementary Figure S6). No differences in leaf anatomy were observed between IR87707-445-B-B-B and IR64 (Supplementary Figure S7). No genetic differences in shoot biomass were observed in the seedling-stage greenhouse studies (Supplementary Table S3).

**Fig. 4.**
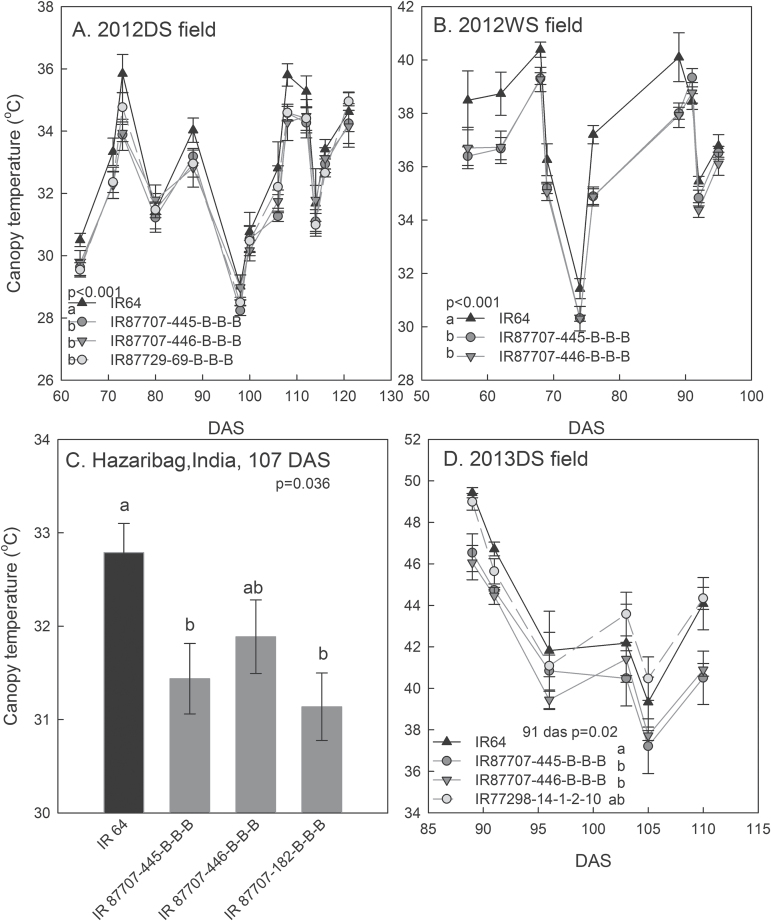
Canopy temperature across four field drought experiments. The canopy temperature was significantly lower in 2-QTL NILs across seasons in (A) 2012DS and (B) 2012WS, and (C) at 107 DAS in Hazaribag, India, and (D) at 91 DAS in 2013DS. The 4-QTL NIL (A) and +QTL BIL (D) also showed lower canopy temperature than IR64. Values shown are mean ± SE, and letters indicate significant differences among genotypes as determined by LSD comparison (*P* < 0.05). *P*-values shown are for genotypic differences across the experiment as determined by the mixed model ASREML. IR64 is indicated by a black line/bar, and the 2-QTL NILs are indicated by dark grey lines/bars.

**Fig. 5.**
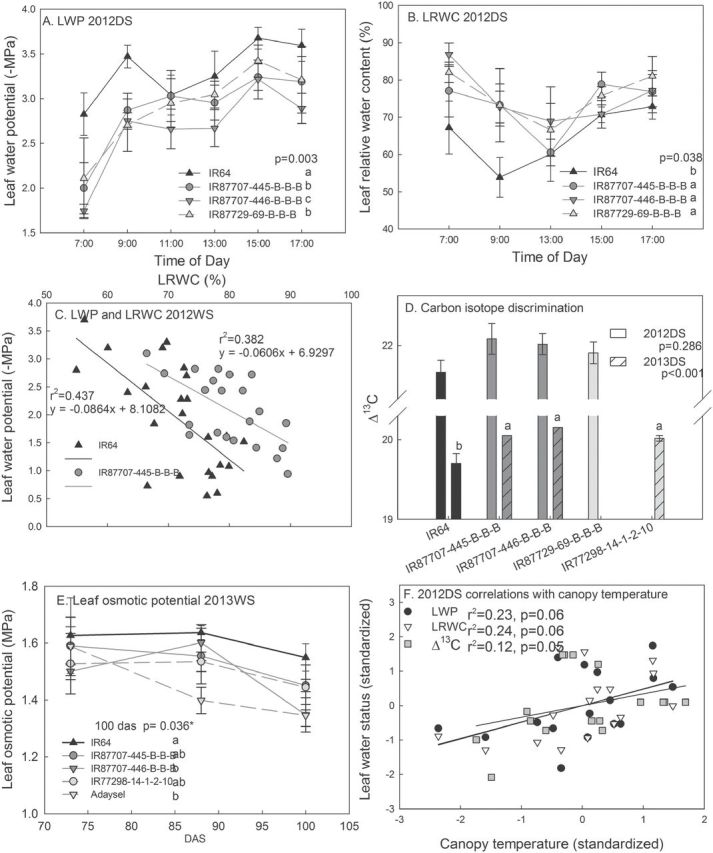
Leaf water status in the drought-stress treatment indicated a greater degree of hydration according to (A) leaf water potential over the course of the day (88 DAS), (B) leaf relative water content over the course of the day (88 DAS), (C) the relationship between LWP and LRWC in rewatered leaves from the field drought treatment that were dehydrated to different levels (90–91 DAS), (D) carbon isotope discrimination (79 DAS in 2012DS, 114 DAS in 2013DS), and (E) leaf osmotic potential. (F) Standardized leaf water status parameters correlated with standardized canopy temperature in 2012DS (sign changed for LRWC values). Values shown are mean ± SE, and letters indicate significant differences among genotypes as determined by LSD comparison (*, *P* < 0.05). *P*-values shown are for genotypic differences across the day (A and B) as determined by the mixed model ASREML. IR64 is indicated by a black line, and the 2-QTL NILs are indicated by dark grey lines in panels A–E.

The BIL and 2-QTL NILs showed consistently lower canopy temperatures than IR64 in the drought treatments in all field experiments (Fig. 4). The 2- and 4-QTL NILs showed consistently less negative LWP and higher LRWC than IR64 across the day in the drought-stress treatment, and re-watered plants of IR87707-445-B-B-B from the drought-stress treatment in the field showed a higher LRWC than IR64 at similar LWP values (Fig. 5A–C). Better leaf water status in the NILs and BILs in the drought-stress treatment was also reflected by higher Δ^13^C values and less negative leaf osmotic potentials than IR64 (Fig. 5D, E; Supplementary Figure S8D). These differences were not observed in the WW treatment (Supplementary Figure S8). Leaf water status parameters showed correlations (*P* = 0.05–0.06) with canopy temperature in 2012DS (LWP, LRWC, Δ^13^C; Fig. 5F), but not for Δ^13^C in 2013DS or osmotic potential in 2013WS.

#### Root morphological parameters: architecture and anatomy

**Fig. 6.**
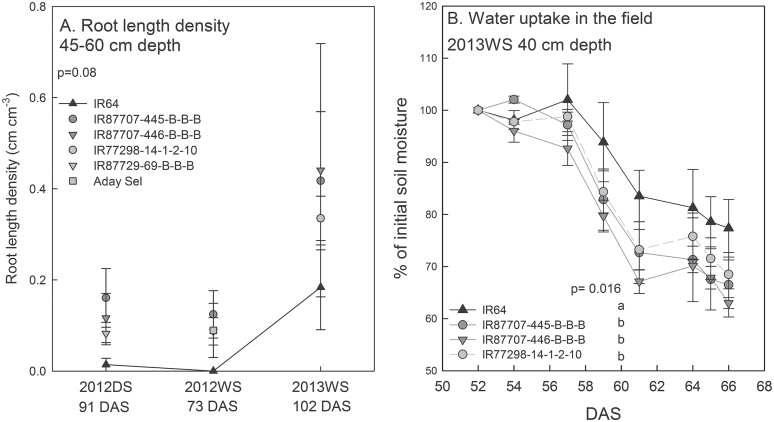
Root length density at 45–60cm depth in the drought treatment of field studies (A), and genotypic differences in soil moisture levels, expressed as percent of initial soil moisture after rewatering (B). Higher soil moisture levels indicate decreased water uptake of IR64. Values shown are mean ± SE, and letters indicate significant differences among genotypes as determined by LSD comparison (*, *P* < 0.05). *P*-values shown are for genotypic differences across the experiment as determined by the mixed model ASREML. IR64 is indicated by a black line, and the 2-QTL NILs are indicated by dark grey lines/symbols.

In general, genetic differences in root morphology were more subtle and growth stage-specific than the differences in the above-ground drought response. In the field drought experiments, root length density (RLD) of the +QTL BIL and the 2-QTL NILs was significantly greater than that of IR64 at the 15–30cm depth in 2013WS, and showed higher but non-significant values in the other experiments and soil depths, which in some experiments showed an RLD of 0 in IR64 at depths below 45cm (2012DS and 2012WS; Fig. 6A; Supplementary Figure S9). This trend was not seen in the well-watered control or in the 2013DS under the low-fertility treatment (Supplementary Figure S9). The maximum root depth (Supplementary Figure S10) and total root length (Supplementary Table S3) in greenhouse experiments did not consistently differ among genotypes under seedling-stage drought stress.

**Fig. 7.**
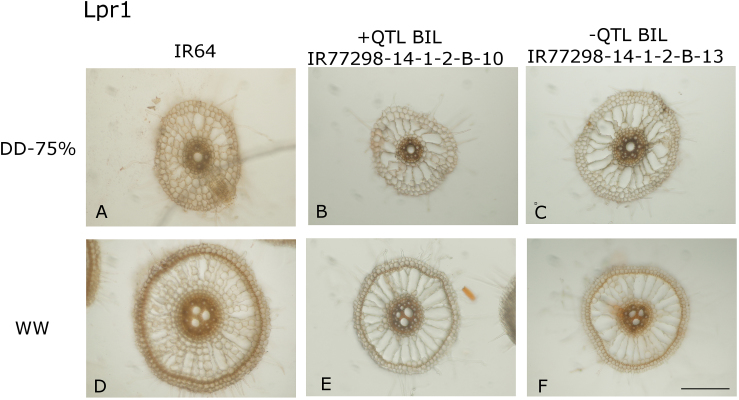
Root anatomy differences between + and –QTL BILs in terms of root and xylem diameter when measured at 2cm from the root tip in greenhouse studies. Images on top are from the DD-75% treatment and images on the bottom are from the WW treatment. Genotypes shown are IR64 (A) DD-75% and (D) WW, +QTL BIL IR77298-14-1-2-10 (B) DD-75% and (E) WW, and –QTL BIL IR77298-14-1-2-13 (C) DD-75% and (F) WW. All images are shown at the same scale, which was a magnification of ×100, and the bar in panel (F) represents 200 µm.

In terms of root anatomy, the +QTL BILs had smaller nodal root and metaxylem vessel diameter than the –QTL BILs near the root apex in the severe-stress (DD-75%) treatment (Lpr1; Table 2, Fig. 7), but not in the WW treatment and not at the longitudinal mid-point of the root (Table 2; Supplementary Table S4). Similar differences were observed between the +QTL BILs and IR64 but were not significant. In contrast, no significant anatomical differences at the root apex between the 2-QTL NILs and IR64 were observed in the greenhouse (Supplementary Table S5), or across depths in the field (Supplementary Table S6).

**Table 2.** Root anatomical parameters from the first greenhouse experiment (Lpr1) on BILs at the apical zone (2cm from the root tip) of 21-day-old seedlings in the DD-75% and WW treatments

Genotype	Root diameter (µm)		Stele diameter (µm)		Metaxylem vessel diameter (µm)		Number of metaxylem vessels		Cortical aerenchyma (%)	
	DD-75%	WW	DD-75%	WW	DD-75%	WW	DD-75%	WW	DD-75%	WW
**Lpr1**										
IR77298-14-1-2-B-10 (+)	291 c	582	107 b	183	26.1 b	31.2	1	3.52	19.7	24.0 bc
IR77298-14-1-2-B-13 (–)	437 ab	540	147 a	168	42.8 a	31.9	1	2.93	21.6	29.2 ab
IR77298-5-6-B-18 (+)	334 bc	661	115 ab	192	32.6 ab	34.5	1	3.64	16.2	32.8 a
IR77298-5-6-B-11(–)	458 a	627	147 a	191	36.6 ab	31.6	1	3.64	14.6	31.0 ab
IR64	326 bc	571	103 b	177	28.3 b	31.2	1	3.05	15	15.9 c

Values shown are means, and those with different letters are significantly different based on ANOVA and LSD comparison.

#### Root functional parameters: water uptake, bleeding rate, and root hydraulic conductivity

Genetic differences in root functional parameters in the field were most evident under the most severe drought-stress treatments at late-vegetative and reproductive stages. The frequency-domain reflectometry sensor measuring soil moisture at a range of soil depths indicated less water uptake by IR64 compared to the +QTL BIL and 2-QTL NILs when expressed as a percentage of the initial reading in 2013WS (Fig. 6B). However, no consistent differences in cumulative water uptake were observed among genotypes under drought over the 3-week growth period in the greenhouse experiments (Supplementary Table S3). Likewise, hourly volumetric soil moisture measurements in the field with sensors at fixed soil depths of 20cm and 40cm did not indicate genotypic differences in water uptake or in diurnal fluctuation/night-time water loss by roots over the 2012WS, 2013DS, and 2013WS seasons (Supplementary Figure 11).

Xylem sap bleeding rate from the intact root zones of field-grown plants was lower in the 4-QTL and 2-QTL NILs compared to IR64 at some stage in all field drought experiments (Supplementary Figure S12), although this was more evident in the dry season than in the wet season and only significant in 2013DS. No consistent genetic differences in bleeding rate were observed in the WW control treatment (Supplementary Figure S12).

**Fig. 8.**
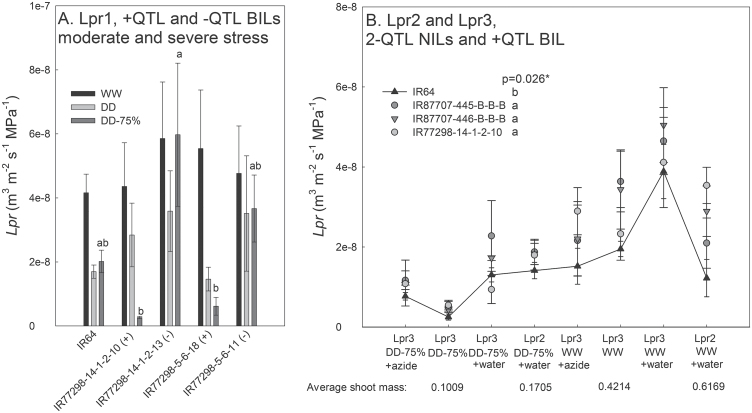
The *Lp*_*r*_ in three studies. Treatments included WW, DD, and DD-75%. (A) Experiment Lpr1 on BILs, in which no plants were rewatered before the *Lp*_*r*_ measurement. (B) Experiments Lpr2 and Lpr3 on 2-QTL NILs and the +QTL BIL: when indicated, plants in the DD-75% treatment were rewatered before the *Lp*_*r*_ measurement (water or azide). Experiments are listed in order of average shoot mass, reflecting the level of drought severity. Values shown are mean ± SE, and letters indicate significant differences among genotypes as determined by LSD comparison (*, *P* < 0.05).

Root hydraulic conductivity responses also showed significant genetic differences. In the Lpr1 experiment comparing +QTL and –QTL BILs, the +QTL BILs showed significantly lower *Lp*_*r*_ than the –QTL BILs under severe drought stress (DD-75%), but not compared to IR64 and not under moderate drought stress (DD) or in the WW control treatment (Fig. 8). In subsequent repeated experiments with the 2-QTL NILs, significantly higher average *Lp*_*r*_ than IR64 was observed in the 2-QTL NILs across experiments and treatments, including in the WW treatment of Lpr2 and in the severe drought-stress treatment of experiment Lpr3 after rewatering and after addition of the aquaporin inhibitor sodium azide (Fig. 8). Although the addition of azide significantly reduced the *Lp*_*r*_ in both treatments of experiment Lpr3, no genetic differences in the degree of inhibition were observed.

## Discussion

The IR64 drought-yield +QTL BILs and NILs in this study had multiple QTL regions from the drought donor AdaySel in common, and both sets of genotypes showed greater yield under drought than recurrent parent IR64. However, different QTL combinations resulted in different levels of improvement over IR64. Multiple drought response mechanisms were observed to be conferred in the highest-yielding QTL combination (*qDTY*_*2.2*_ + *qDTY*_*4.1*_), and these appear to be related to improved transpiration. The performance of the 2-QTL NILs points to the complex interaction among major-effect drought-yield QTLs that do not necessarily act to complement each other when collectively present in a common genetic background.

The pattern of effect of *qDTY*_*2.2*_ and *qDTY*_*4.1*_ across different stress levels showed the effect of *qDTY*_*2.2*_ to be specific to the most severe stress conditions, while *qDTY*_*4.1*_ showed a higher effect under milder stress conditions. The two QTLs were originally identified in experiments with different levels of stress severities in the IR77298-14-1-2 × IR64 population (Swamy *et al.*, 2013). However, the present study attempts to show a pattern of effect of these QTLs across seven different experiments conducted under varying severities of stress. The rainfed rice environment is highly variable in terms of stress severity across seasons and areas; the distinct effect patterns shown by *qDTY*_*2.2*_ and *qDTY*_*4.1*_ indicate that their combination could be complementary to achieve yield advantages under a wider range of stress severities.

The different levels of drought response among NILs with different QTL combinations, particularly those with only *qDTY*_*2.2*_ and *qDTY*_*4.1*_, are in agreement with the results reported by Dixit *et al.* (2012) where QTL peaks at slightly different chromosomal locations were detected within the QTL regions of *qDTY*_*9.1*_ and *qDTY*_*12.1*_ depending on the level of drought stress severity. Furthermore, Dixit *et al.* (2012) detected a sub-region of *qDTY*_*9.1*_ that was related to a detrimental effect on yield, which is reflected here in the lesser degree of improvement of lines with *qDTY*_*9.1*_ in terms of canopy temperature and NDVI under drought (Fig. 3). However, further characterization of *qDTY*_*9.1*_ and *qDTY*_*10.1*_ across a range of drought-stress severities is needed to understand why the 4-QTL NILs did not perform as well as the 2-QTL NILs (*qDTY*_*2.2*_ and *qDTY*_*4.1*_) under drought. *qDTY*_*2.2*_ has also been reported to show an effect on grain yield under upland reproductive-stage drought stress in the background of variety MTU1010 with KaliAus as the donor parent (Sandhu *et al.*, 2014), although the physiological mechanisms behind the yield advantage in that case have not yet been characterized. More research is necessary to know if *qDTY*_*2.2*_ and *qDTY*_*4.1*_ confer similar mechanisms in other backgrounds to those observed in the IR64 background.

Yield under drought is affected by T (transpiration), TE (transpiration efficiency), and HI (harvest index) (Passioura, 1977). The traits observed to be conferred by the 2-QTL NILs in this study appear to be related to T, but not to TE or HI. Under drought, the 2-QTL NILs showed improved leaf water status (as evidenced by canopy temperature, LWP, LRWC, leaf osmotic potential, and Δ^13^C). The significantly lower canopy temperatures observed here, and those previously reported in the +QTL BILs (Swamy *et al.*, 2013), as well as less negative LWP, higher RWC, and higher Δ^13^C (indicating lower transpiration efficiency possibly due to more open stomata) all indicated higher levels of leaf hydration and transpiration under drought. This improved leaf water status under drought appears to be a consequence of the causal mechanisms affecting the amount of useable water: higher root hydraulic conductivity, and in some cases higher root length density at depth. The slightly higher biomass of the 2-QTL NILs and higher Δ^13^C also indicated that TE was not related to yield under drought in the 2-QTL NILs, and a lack of difference in HI in well-watered conditions further points to T as the main factor behind yield under drought of the 2-QTL NILs.

Combining drought response traits may increase the level of stability in grain yield improvement across growing seasons. In this study, we observed multiple root traits to be affected by introgressing AdaySel-derived drought-yield QTLs into IR64. The single QTL NILs and the 2-QTL NILs both showed lower canopy temperature and higher NDVI than IR64, and both root traits (increased RLD at depth and increased *Lpr*) could result in lower canopy temperature and improved plant growth under drought. Therefore, further characterization of the single-QTL NILs is necessary to understand whether the strong effects on yield under drought of *qDTY*_*2.2*_ and *qDTY*_*4.1*_ are due to additive effects the of the QTL on both root traits or complementarity of distinct traits conferred by the two QTLs.

It was expected that the +QTL BILs and NILs would have achieved improved leaf hydration via increased water uptake, most likely through greater root length at depth. However, no differences were observed in water uptake or root growth in the greenhouse seedling experiments or in the field, except in 2013WS when only the sensor measuring soil moisture at multiple depths detected genetic differences. Although RLD at depth was greater in the NILs and +QTL BIL in some field experiments presented here, we previously reported that RLD at depth was not significantly greater in the +QTL BILs when grown in soil with higher clay content (about 50%; Swamy *et al.*, 2013) and with a longer history of puddling than the soil in the present study (about 40% clay). Therefore, it appears that soil type better explains the differences in root growth between the present study and that observed previously, rather than generation advancement or QTL combinations.

Several factors may have contributed to the stronger differences in leaf water status than in water uptake/root growth observed here: (i) the methods used for water uptake and root growth measurements may be more prone to experimental error caused by the heterogeneous soil environment compared with above-ground measurements; (ii) it may be that subtle differences below ground can have large effects on above ground plant water status under drought; and (iii) other factors affecting leaf water status, particularly root hydraulics, may also have contributed to the improved drought response of the +QTL BILs and NILs compared with IR64.

Hydraulic properties have been implicated in the drought response of other crops, particularly with respect to conservative water uptake allowing more water to be available during the critical reproductive stage, through stomatal regulation mechanisms as well as root anatomical and functional parameters (Richards and Passioura, 1989; Zaman-Allah *et al.*, 2011; Schoppach *et al.*, 2013). In our study, the water uptake, gas exchange, and carbon isotope discrimination results indicate that these NILs did not exhibit conservative water uptake and did not have greater water use efficiency conferred by stomatal control. However, the +QTL BILs showed significantly smaller root and xylem vessel diameters than the –QTL BILs (Table 2; Fig. 7), which had larger root and xylem vessel diameters than IR64.

Small root xylem vessel diameter affects axial water transport according to Poisseuille’s law (conductivity is proportional to vessel diameter to the fourth power) and has been reported to result in conservative water uptake in wheat (Richards and Passioura,1989; Schoppach *et al.*, 2013). In rice, we have observed traditional drought-resistant varieties responding to drought stress by decreasing the xylem vessel diameter near the root apex, and this response was not observed in IR64 (Henry *et al.*, 2012). In addition to restricting water uptake, smaller xylem vessels may be beneficial in rice under drought since rice has been reported to be susceptible to xylem vessel cavitation caused by drought stress (Stiller *et al.*, 2003), and smaller xylem vessels may be less likely to cavitate under high evapo-transpiration demand (Hacke and Sperry, 2001). However, other rice studies conclude that large xylem vessel diameter may be beneficial for improving the drought response; larger root diameters have been associated with drought resistance in OsNAC10 and OsNAC5 transgenics (Jeong *et al*., 2010, 2012). In this study, the genotypes with small xylem vessel diameters (+QTL BILs) were those that yielded more under drought than those with larger xylem vessel diameters (–QTL BILs), supporting the former perspective. However, this trait was not associated with yield under drought in the best-performing lines in the study (the 2-QTL NILs). Furthermore, the significantly lower canopy temperature of the +QTL BILs compared to the –QTL BILs and IR64 (Swamy *et al.*, 2013) suggests that the differences in xylem vessel diameter at the root tip may have had only a minor effect on the overall plant water status under drought in the field.

Generation advancement to NILs revealed the 2-QTL NILs to show generally higher *Lpr* than IR64 across experiments and treatments (Fig. 8). Although few differences in total water uptake were observed, these differences in *Lpr* point to improved root function in the 2-QTL NILs, but this appears to be independent of treatment, root anatomy, and azide-responsive aquaporins. Although previous microarray analysis did not identify aquaporin genes to be differentially expressed within the QTL regions in the +QTL BILs (Moumeni *et al.*, 2011; Swamy *et al.*, 2013), a role of aquaporins may not have been detected since aquaporin response could be related to protein modification rather than expression (Chaumont and Tyerman, 2014). More research on aquaporin protein structure of the 2-QTL NILs and experiments with additional aquaporin inhibitors may reveal more-detailed mechanisms behind the higher *Lpr* of the 2-QTL NILs.

Aquaporins have previously been implicated in differential transpiration and conservative water uptake function between drought resistant and susceptible genotypes in soybean (Sadok and Sinclair, 2010), peanut (Devi *et al.*, 2012), wheat (Schoppach *et al.*, 2013), and sorghum (Choudhary *et al.*, 2013). In contrast, we observed trends of higher *Lp*_*r*_ and no evidence of conservative water uptake in the lines showing highest yield under drought in this study. These results, and the fact that rice typically shows reduced ability to draw down soil moisture levels compared to other crops (Kondo *et al.*, 2000), imply that rice might be different from other crops in terms of ideal hydraulic properties for improved response to drought; it may be increased water uptake, rather than conservative water uptake, that could improve the drought response of lowland rice varieties like IR64.

Interestingly, the sap bleeding rate from root zones of the +QTL BILs and 2-QTL NILs did not correlate with the trends seen for *Lp*_*r*_. Root pressure (potentially indicated by sap bleeding rate) has been hypothesized as important for the rice drought response as a mechanism for refilling of xylem embolism (Lafitte and Courtois, 2002). More research is necessary to understand the relationship between drought resistance and sap bleeding rate, as well as the relationships between sap bleeding rate with *Lp*_*r*_, leaf water status, and environmental conditions.

## Conclusions

Using improved lowland rice genotypes that were more than 95% genetically similar to the drought-susceptible parent IR64, this study aimed to characterize the physiological mechanisms behind the drought response conferred by AdaySel-derived QTLs. Greater differences from IR64 in terms of yield, canopy temperature, and NDVI were seen in lines with two QTLs (*qDTY*_*2.2*_ and *qDTY*_*4.1*_) introgressed, rather than with three or four QTLs introgressed together. Under drought stress, we observed the improved genotypes to have higher root hydraulic conductivity and in some cases higher root length density at depth, resulting in better leaf water status (as evidenced by canopy temperature, LWP, LRWC, leaf osmotic potential, and Δ^13^C). Consistent with the trends in yield advantage of the QTLs under drought, the drought resistance traits associated with these QTLs were most strongly expressed under severe drought stress, although the two QTLs each showed maximum effects at different stress severities. These results highlight the complex interactions among major-effect drought-yield QTLs, and the need to find the optimal combination of QTLs that act to complement each other when present in a common genetic background. Given the difficulties in detecting significant differences among genotypes for many of the traits measured in the field experiments, more-detailed measurements in controlled conditions could contribute to a better understanding of the physiological mechanisms behind *qDTY*_*2.2*_ and *qDTY*_*4.1*_.

## Supplementary material

Supplementary data can be found at *JXB* online.

Supplementary Table S1. Soil chemical and physical properties in the field and greenhouse experiments.

Supplementary Table S2. Ambient conditions in the field and greenhouse experiments.

Supplementary Table S3. Plant growth in seedling-stage greenhouse studies.

Supplementary Table S4. Root anatomical parameters at the mid-point of nodal roots from the Lpr1 greenhouse experiment.

Supplementary Table S5. Root anatomical parameters from greenhouse experiments at the apical zone (2cm from the root tip) of 21-day-old seedlings in the DD-75% and WW treatments.

Supplementary Table S6. Root anatomical parameters from the 2012DS field experiment at three soil depths in the stress and WW control treatments.

Supplementary Figure S1. Soil water potential at a depth of 30cm across each of the six field drought experiments.

Supplementary Figure S2. Shoot biomass in the drought-stress treatments across seasons.

Supplementary Figure S3. Shoot biomass in the WW treatments across seasons.

Supplementary Figure S4. Leaf area in the drought-stress and WW treatments across seasons.

Supplementary Figure S5. NDVI in the drought-stress treatments across seasons.

Supplementary Figure S6. Photosynthesis rates on selected dates in 2012 and 2013.

Supplementary Figure S7. Leaf anatomy of IR87707-445-B-B-B and IR64.

Supplementary Figure S8. Leaf water status in the WW treatment according to LWP over the course of the day, carbon isotope discrimination, and leaf osmotic potential. Also leaf osmotic potential in the Lpr3 seedling-stage greenhouse study.

Supplementary Figure S9. Distribution of root length density with depth in the drought-stress and well-watered treatments of the field studies.

Supplementary Figure S10. Maximum root depth in greenhouse studies.

Supplementary Figure S11. Trends in soil moisture across the seasons 2012WS, 2013DS, and 2013WS and diurnal trends.

Supplementary Figure S12. Xylem sap bleeding rate from the root zones of field-grown plants.

## Funding

This work was funded by the Generation Challenge Programme project G3008.06 ‘Targeting Drought-Avoidance Root Traits to Enhance Rice Productivity under Water-Limited Environments’ and the Bill & Melinda Gates Foundation project ‘Stress-Tolerant Rice for Africa and South Asia’.

## Supplementary Material

Supplementary Data
