# Supplementary material for: Physiological mechanisms contributing to the QTL-combination effects on improved performance of IR64 rice NILs under drought
Source: J Exp Bot. 2015 Feb 13;66(7):1787–99. doi: 10.1093/jxb/eru506 (PMC4378621; doi:10.1093/jxb/eru506)
Supplement: Supplementary Data [file supp_66_7_1787__index.html]

Physiological mechanisms contributing to the QTL-combination effects on improved performance of IR64 rice NILs under drought — Physiological mechanisms contributing to the QTL-combination effects on improved performance of IR64 rice NILs under drought — Supplementary Data 

# Physiological mechanisms contributing to the QTL-combination effects on improved performance of IR64 rice NILs under drought

## Supplementary Data

Data files

**Files in this Data Supplement:**

- Supplementary Data - Supplementary Data
