# Supplementary material for: Physiological mechanisms contributing to the QTL-combination effects on improved performance of IR64 rice NILs under drought
Source: J Exp Bot. 2015 Feb 13;66(7):1787–99. doi: 10.1093/jxb/eru506 (PMC4378621; doi:10.1093/jxb/eru506)
Supplement: Supplementary Data [file supp_eru506_jexbot127175_file001.pdf]

Henry et al. “Physiological mechanisms contributing to the QTL-combination effects on improved performance of IR64 rice NILs under drought”

Supplemental Material

Supp. Table 1. Soil chemical and physical properties in the field and greenhouse experiments.

	Avail. P (mg kg^{-1})	Exch. K (meq $100g^{-1}$)	Exch. Mg (meq $100g^{-1}$)	Exch. Ca (meq $100g^{-1}$)	Kjeldahl N (%)	pH	Clay (%)	Sand (%)	Silt (%)
Field experiments (season)									
2011WS	27	1.49	9.7	17.1	0.121	-	38	21	41
2012DS, 2012WS, 2013WS	6.2	38.0	1.22	14.0	0.111	-	-	-	-
Hazaribag, India	12.6	0.93	-	-	-	7.66	34	30	36
2013DS control	10	1.02	10.3	15.4	0.106	6.4	42	16	42
2013DS stress	20	1.62	13.0	19.1	0.112	7.4	39	18	43
Greenhouse experiments									
Lpr1	70.7	-	-	-	0.261	5.77	-	-	-
Lpr2	38	1.22	6.2	14	0.111		34	19	37
Lpr3	48	1.32	7.04	14.5	0.108	6.2	36	15	49
AdaySel4	42	1.52	6.43	13.1		6.2	35	17	48

Supp. Table 2. Ambient conditions in the field and greenhouse experiments.

	Min. temp (°C)	Max. temp (°C)	Ave. temp (°C)	Ave. RH (%)	Average daily solar radiation (MJ m^{-2})
Field experiments (season)					
2011WS	22.2	34.7	27.7	87.3	14.0
2012DS	20.9	35.1	27.0	86.2	15.3
2012WS	21.5	35.5	27.6	89.5	13.6
Hazaribag, India[a]	8.5	42.0	-	74.5	-
2013DS	19.9	35.8	27.1	82.6	16.3
2013WS	21.7	33.9	27.7	86.6	13.3
Greenhouse experiments					
Lpr1	24.8	49	31.1	71.7	15.5[b]
Lpr2	22.6	45.6	30.0	63.1	13.7[b]
Lpr3	24.0	48.1	32.1	60.9	16.2[b]
AdaySel4	25.2	48.5	30.8	60.4	12.0[b]

[a]These values were measured outside the rainout shelter.
[b]These values were measured outside the greenhouse.

Supp. Table 3. Plant growth in seedling stage greenhouse studies. Values shown are means ± s.e. Values with different letters are significantly different.

Genotype	Shoot mass (g)			Total root length (cm)			Total water uptake (g)		
	WW	DD-75%	DD	WW	DD-75%	DD	WW	DD-75%	DD
**A) Lpr1**									
IR77298-14-1-2-B-10 (+)	0.546	0.109	0.248	1010	637	1045	201	36.9	87
IR77298-14-1-2-B-13 (-)	0.357	0.122	0.248	837	429	1243	171	34.2	93
IR77298-5-6-B-18 (+)	0.522	0.079	0.308	1824	536	1743	194	36.0	104
IR77298-5-6-B-11(-)	0.525	0.102	0.237	1252	496	1290	198	35.9	79
IR64	0.452	0.086	0.273	1323	637	1487	194	36.9	91
**B) Lpr2**									
IR77298-14-1-2-B-10 (4)	0.633	0.178		4502 b	1691		396	115	
IR87707-445-B-B-B (2)	0.655	0.200		4738 a	1542		397	128	
IR87707-446-B-B-B (2)	0.609	0.138		4635 ab	1635		373	104	
IR64	0.571	0.166		4528 b	2080		364	122	
**C) Lpr3**									
IR77298-14-1-2-B-10 (4)	0.458	0.103		5642	1831		390	94.1	
IR87707-445-B-B-B (2)	0.410	0.078		5260	1483		342	85.1	
IR87707-446-B-B-B (2)	0.388	0.123		4831	1673		351	96.1	
IR64	0.414	0.078		5296	1788		387	92.6	
**D) Root anatomy4**									
IR77298-14-1-2-B-10 (4)	0.645 a	0.194		-	-		256 a	56.9	
IR87707-445-B-B-B (2)	0.476 c	0.133		-	-		181 b	44.4	
AdaySel	0.526 bc	0.165		-	-		234 ab	50.0	
IR64	0.588 ab	0.159		-	-		283 a	49.6	

Supp. Table 4. Root anatomical parameters at the mid-point of nodal roots from the Lpr1 greenhouse experiment.

Genotype	Root diameter (μm)	Stele diameter (% of root diam)	Metaxylem vessel diameter (μm)	Number of metaxylem vessels	% Cortical Aerenchyma
			**DD-75% mid-point**		
**IR77298-14-1-2-10 (+)**	43.4 ab	32.9 ab	3.11 ab	1.31	22.4
**IR77298-14-1-2-13 (-)**	47.8 ab	30.5 a	3.70 a	1.16	24.2
**IR 77298-5-6-18 (+)**	27.2 b	34.3 a	2.22 b	1.05	18.8
**IR77298-5-6-11(-)**	37.3 ab	32.0 ab	3.01 ab	1.15	23.6
**IR 64**	51.5 a	32.1 ab	3.27 ab	1.31	23.9
			**WW mid-point**		
**IR77298-14-1-2-10 (+)**	72.6	33.0	3.74 ab	3.72	43.1
**IR77298-14-1-2-13 (-)**	55.1	31.5	3.37 b	3.68	35.7
**IR 77298-5-6-18 (+)**	61.5	32.3	4.02 a	3.42	36.7
**IR77298-5-6-11(-)**	76.0	33.7	4.05 a	3.77	28.4
**IR 64**	63.4	32.7	3.47 b	3.36	39.8

Supp. Table 5. Root anatomical parameters from greenhouse experiments at the apical zone (2 cm from the root tip) of 21-day-old seedlings in the drydown from 75% of field capacity (DD-75%) and well-watered (WW) treatments. Values shown are means, and those with different letters are significantly different.

Genotype	Root diameter (μm)		Stele diameter (μm)		Metaxylem vessel diameter (μm)		Number of metaxylem vessels		% Cortical aerenchyma	
	DD-75%	WW	DD-75%	WW	DD-75%	WW	DD-75%	WW	DD-75%	WW
**Lpr2**										
IR77298-14-1-2-B-10 (4)	349 ab	805	123 b	225	27.5 b	39	1.33	4.04	14.1	13.3
IR87707-445-B-B-B (2)	454 a	717	156 a	207	32.8 a	37.8	1.6	4.03	19.8	18.1
IR87707-446-B-B-B (2)	374 ab	727	143 ab	207	31.3 ab	38.2	1.3	4.21	17.1	13
IR64	367 b	730	125 b	214	31.7 ab	37.4	1.03	3.95	16.7	10.7
**Lpr3**										
IR77298-14-1-2-B-10 (4)	550	784	186	189	31.8 a	35.9	1.04	4.51	19.1	17.8 b
IR87707-445-B-B-B (2)	553	767	173	197	27.5 b	36.8	1.02	4.57	21.1	17.9 b
IR87707-446-B-B-B (2)	429	776	135	183	29.1 ab	35.1	1.13	4.47	19.6	16.8 b
IR64	503	748	166	179	27.3 b	36.5	1.22	4.20	19.8	21.3 a
**Root anatomy4**										
IR77298-14-1-2-B-10 (4)	354	678 ab	121	202	32.6	35.0	1.20	3.85	18.4	22.5
IR87707-445-B-B-B (2)	318	646 b	111	194	27.8	34.4	1.00	3.96	21.8	23.1
AdaySel	338	719 a	123	211	29.3	36.7	1.20	4.06	18.7	24.5
IR64	354	715 a	119	208	29.5	36.9	1.39	3.66	16.3	22.8

Supp. Table 6. Root anatomical parameters from the 2012DS field experiment at three soil depths in the stress and well-watered control treatments. Values shown are means, and those with different letters are significantly different.

		**Whole root diameter (μm)**		**Stele diameter (μm)**		**Metaxylem vessel diameter (μm)**		**Number of metaxylem vessels**	
**Depth (cm)**	**Genotype**	**Stress**	**Control**	**Stress**	**Control**	**Stress**	**Control**	**Stress**	**Control**
**15-30**	IR64	279	329	68	71	14.9	27.4	2.24	3.23
	IR87707-445-B-B-B	323	358	77	75	15.2	28.7	2.88	3.38
	IR87707-446-B-B-B	336	303	80	66	15.1	24.5	3.16	3.20
**30-45**	IR64	345	727	87	189	21.9	33.9	1.28	4.17 a
	IR87707-445-B-B-B	391	597	101	152	24.9	25.8	1.53	3.43 a
	IR87707-446-B-B-B	437	567	104	140	26.9	25.4	1.44	2.64 b
**45-60**	IR64	397	568	129	149	59.7	57.9	1.40	3.24
	IR87707-445-B-B-B	557	582	126	140	53.0	56.4	2.15	2.92
	IR87707-446-B-B-B	621	609	149	166	57.9	70.8	2.50	2.61

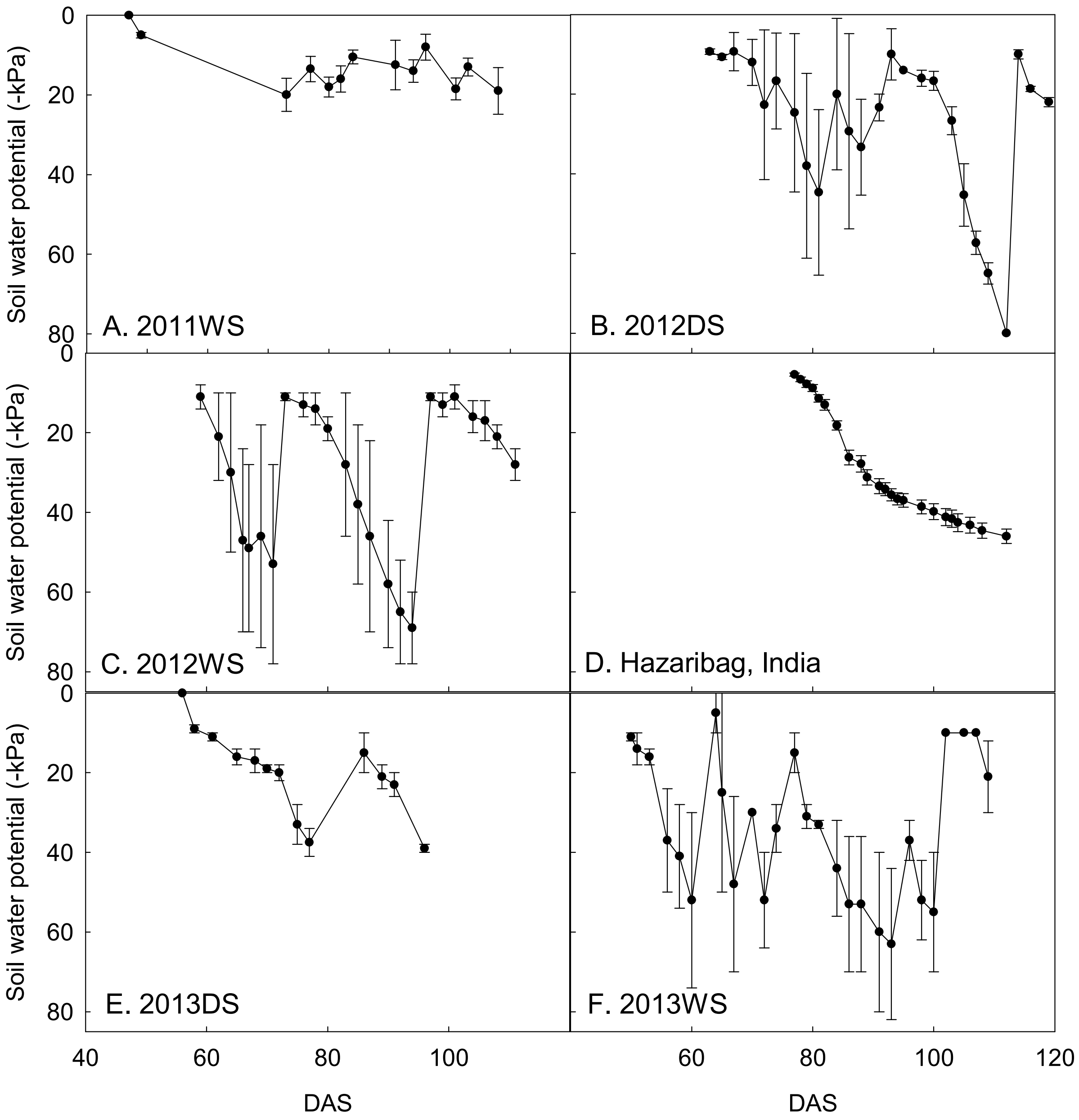

Supp. Fig. 1. Soil water potential at a depth of 30 cm across each of the 6 field drought experiments. Values shown are means of 2-5 tensiometers ± se.

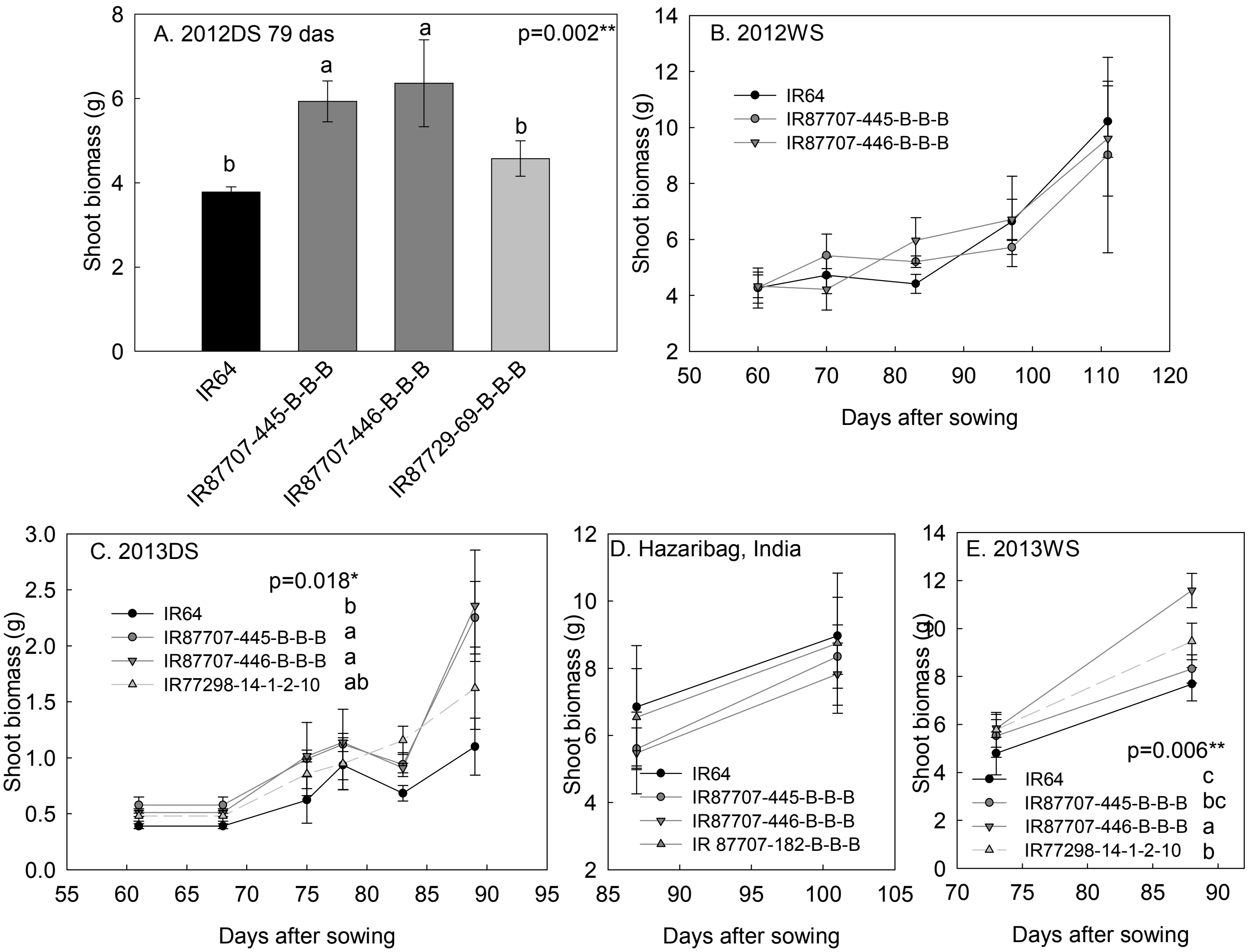

Supp. Fig. 2. Shoot biomass in the drought stress treatments across seasons. P-values shown are for genotypic differences across the experiment as determined by the mixed model ASREML. IR64 is indicated by a black line/bar, and the 2-QTL NILs are indicated by dark gray lines/bars.

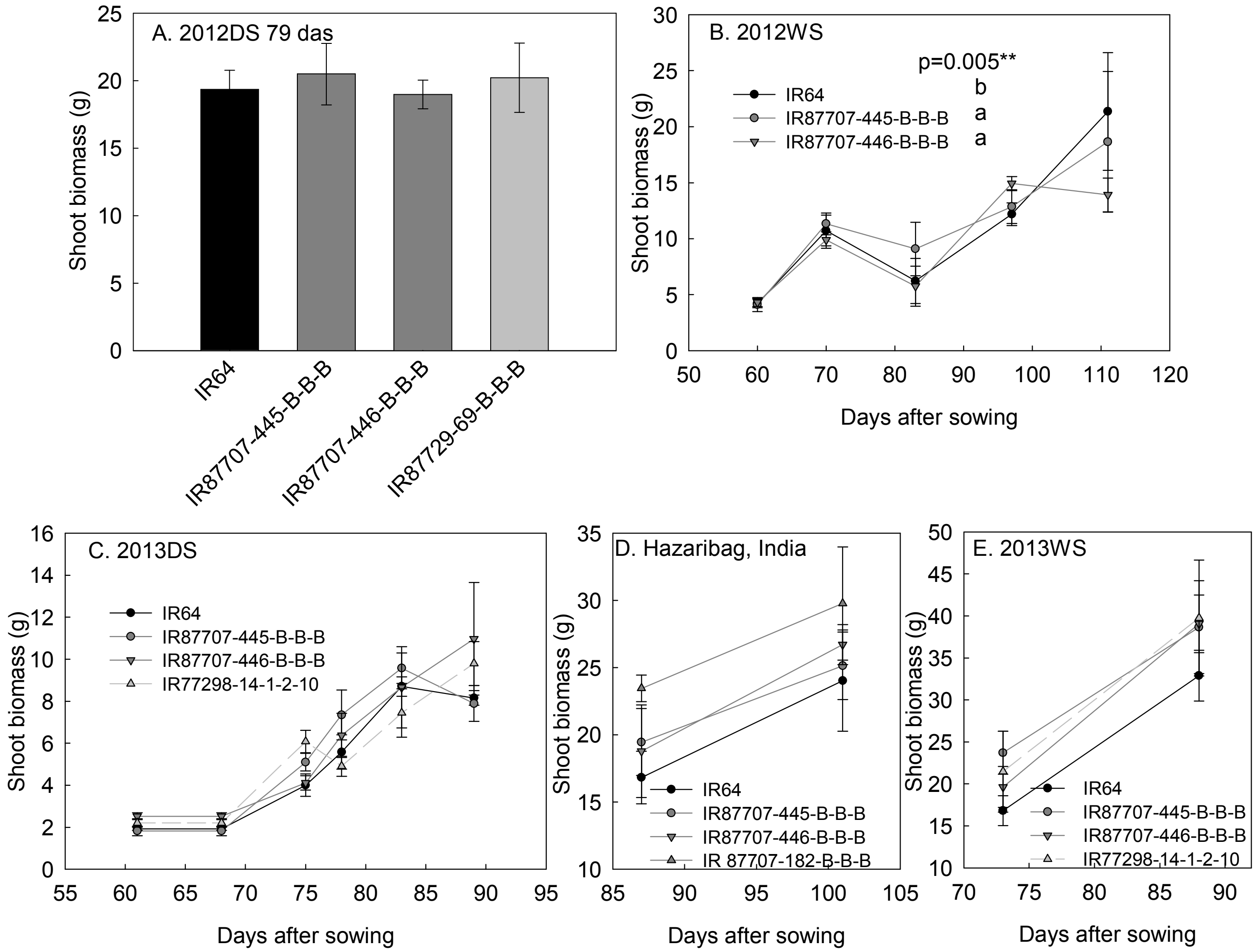

Supp. Fig. 3. Shoot biomass in the well-watered treatments across seasons. Except for 2012WS, no significant differences in shoot biomass were observed among genotypes in the well-watered treatments. IR64 is indicated by a black line/bar, and the 2-QTL NILs are indicated by dark gray lines/bars.

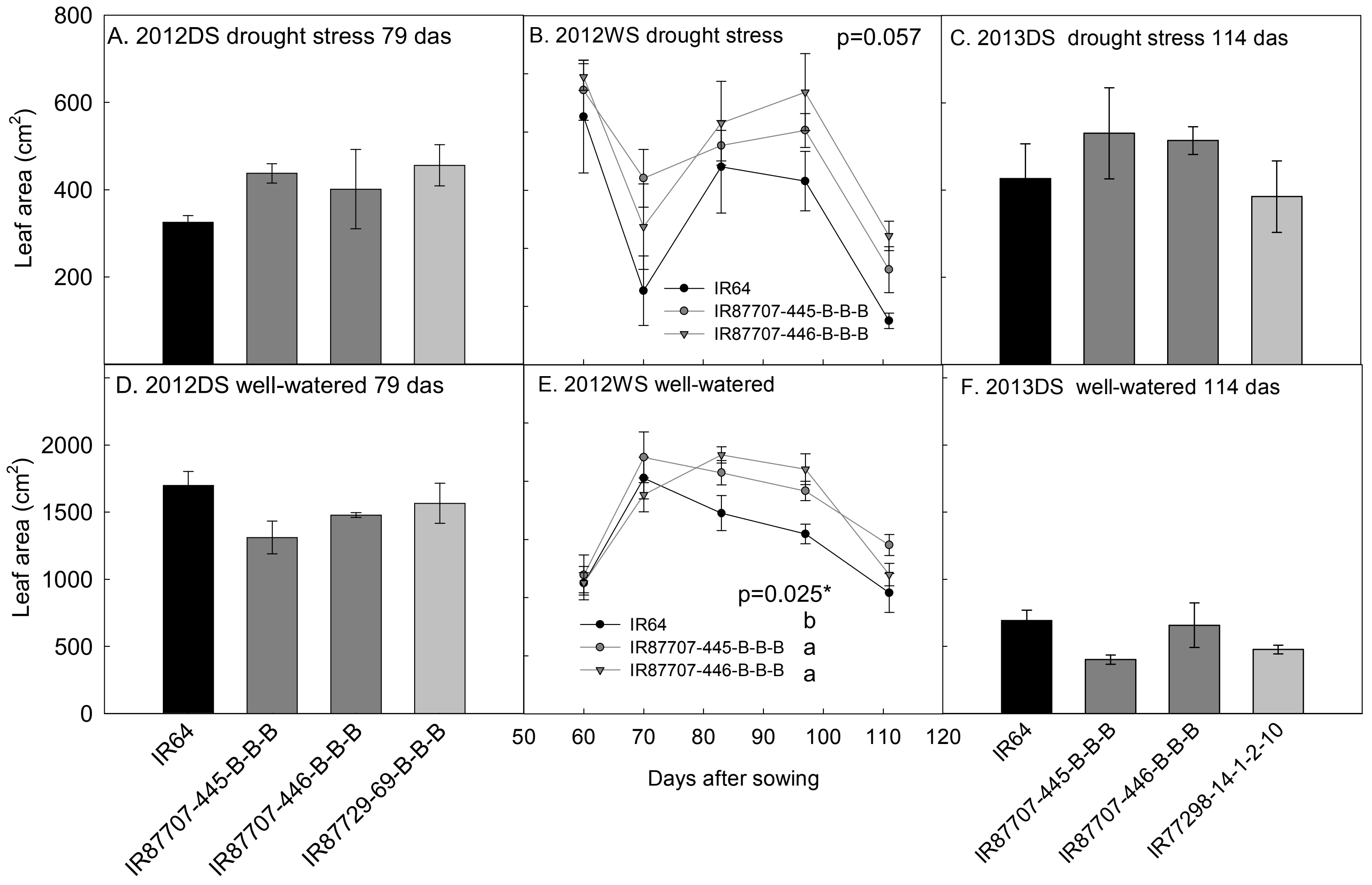

Supp. Fig. 4. Leaf area in the drought (A-C) and well-watered (D-F) treatments across seasons. P-values shown are for genotypic differences across the experiment as determined by the mixed model ASREML. IR64 is indicated by a black line/bar, and the 2-QTL NILs are indicated by dark gray lines/bars.

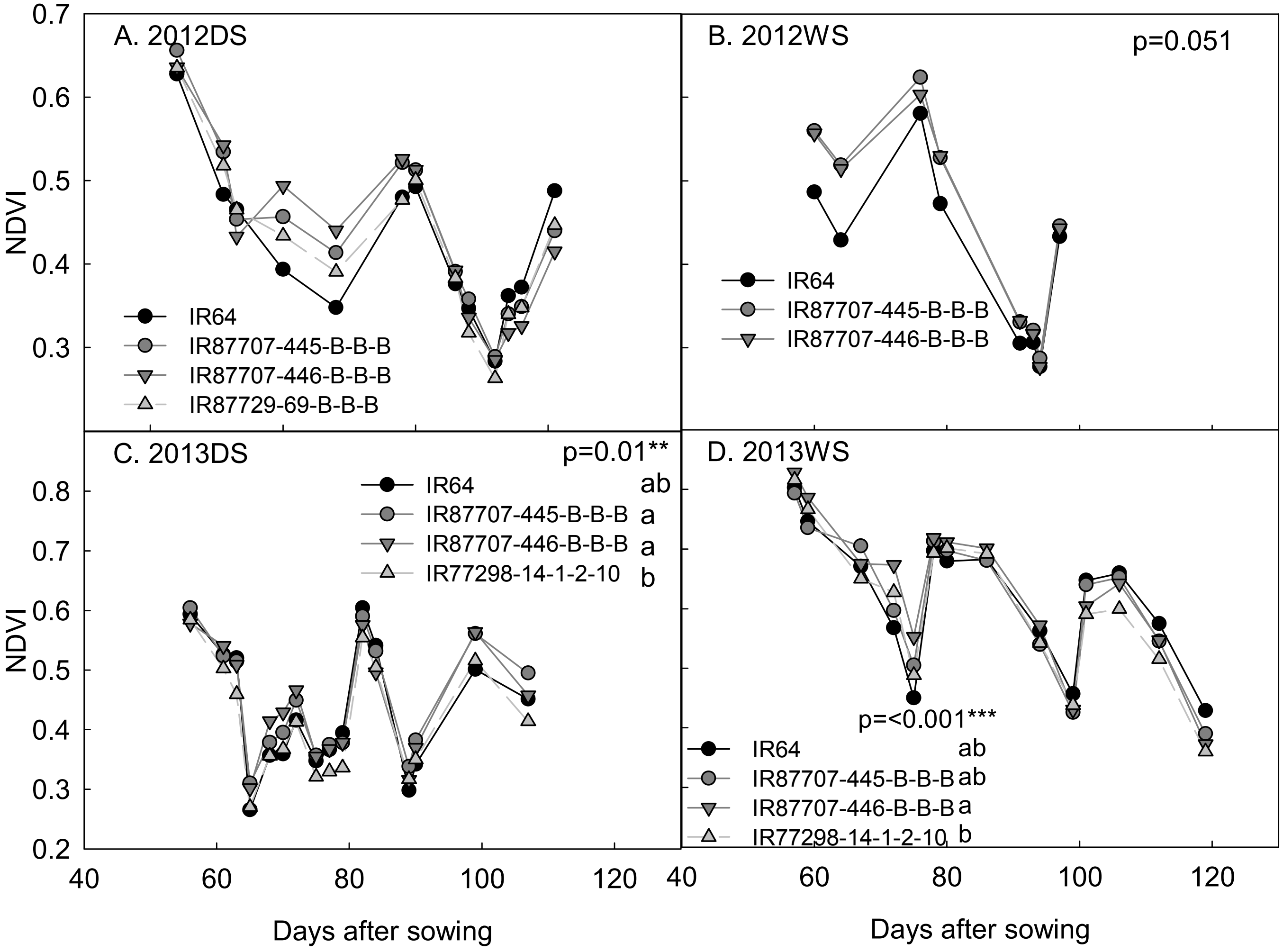

Supp. Fig. 5. Normalized difference vegetation index (NDVI) in the drought treatments across seasons. P-values shown are for genotypic differences across the experiment as determined by the mixed model ASREML. IR64 is indicated by a black line, and the 2-QTL NILs are indicated by dark gray lines.

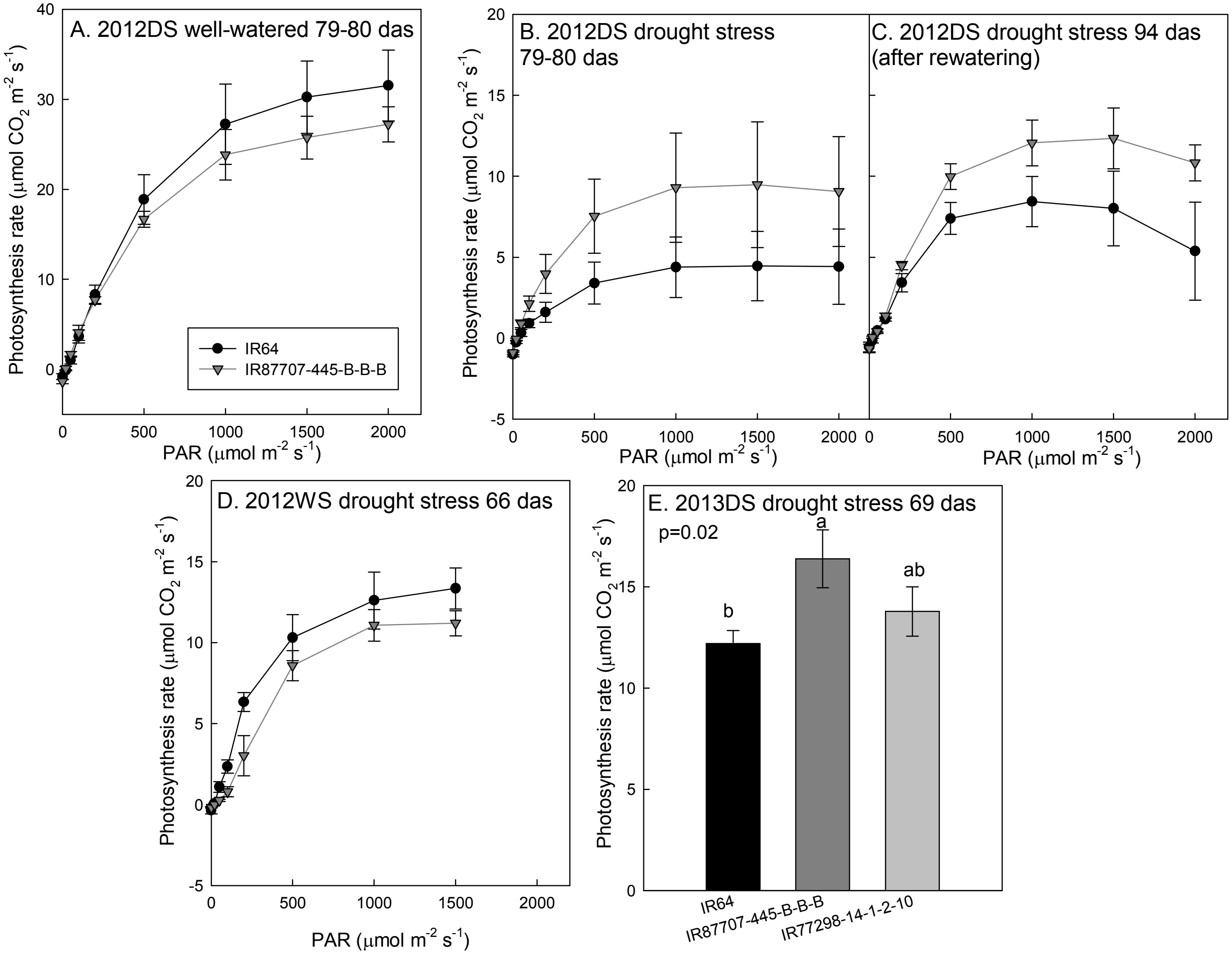

Supp. Fig. 6. Photosynthesis rates on selected dates in 2012 and 2013. Significant differences among genotypes were observed in the 2013DS experiment (E) only. IR64 is indicated by a black line/bar, and the 2-QTL NIL is indicated by dark gray lines/bars.

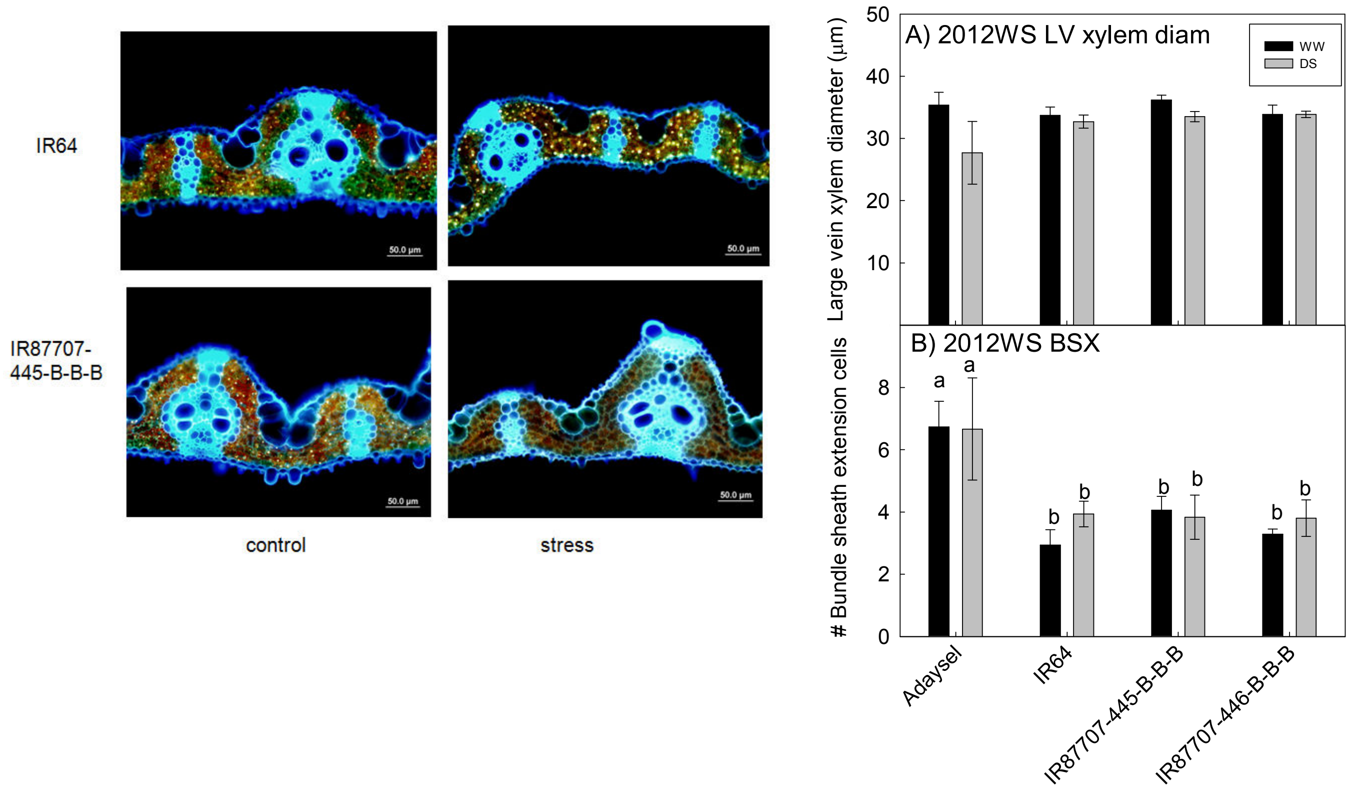

Supp. Fig. 7. Leaf anatomy of IR87707-445-B-B-B and IR64. A) example leaf sections; B) example anatomical parameters in which leaf anatomy was similar among genotypes. The total parameters measured were: MCNO: mesophyll cell number, MCWD: mesophyll cell width, MCTA: mesophyll cell total area, SVWD: small vein width, SVHT: small vein height, SV-LFTH: small vein leaf thickness, IVD-SV: interveinal distance small vein, SV-BSCN: small vein bundle sheath cell number, SV-BSCA: small vein bundle sheath cell area, LVWD: large vein width, LVHT: large vein height, LV-LFTH: large vein leaf thickness, IVD-LV: interveinal distance large vein, LV-BSCN: large vein bundle sheath cell number, LV-BSCA: large vein bundle sheath cell area, #LV xylem number: of large vein xylem vessels, LV xylem diam: large vein xylem vessel diameter, and #BSX: number of bundle sheath extension cells.

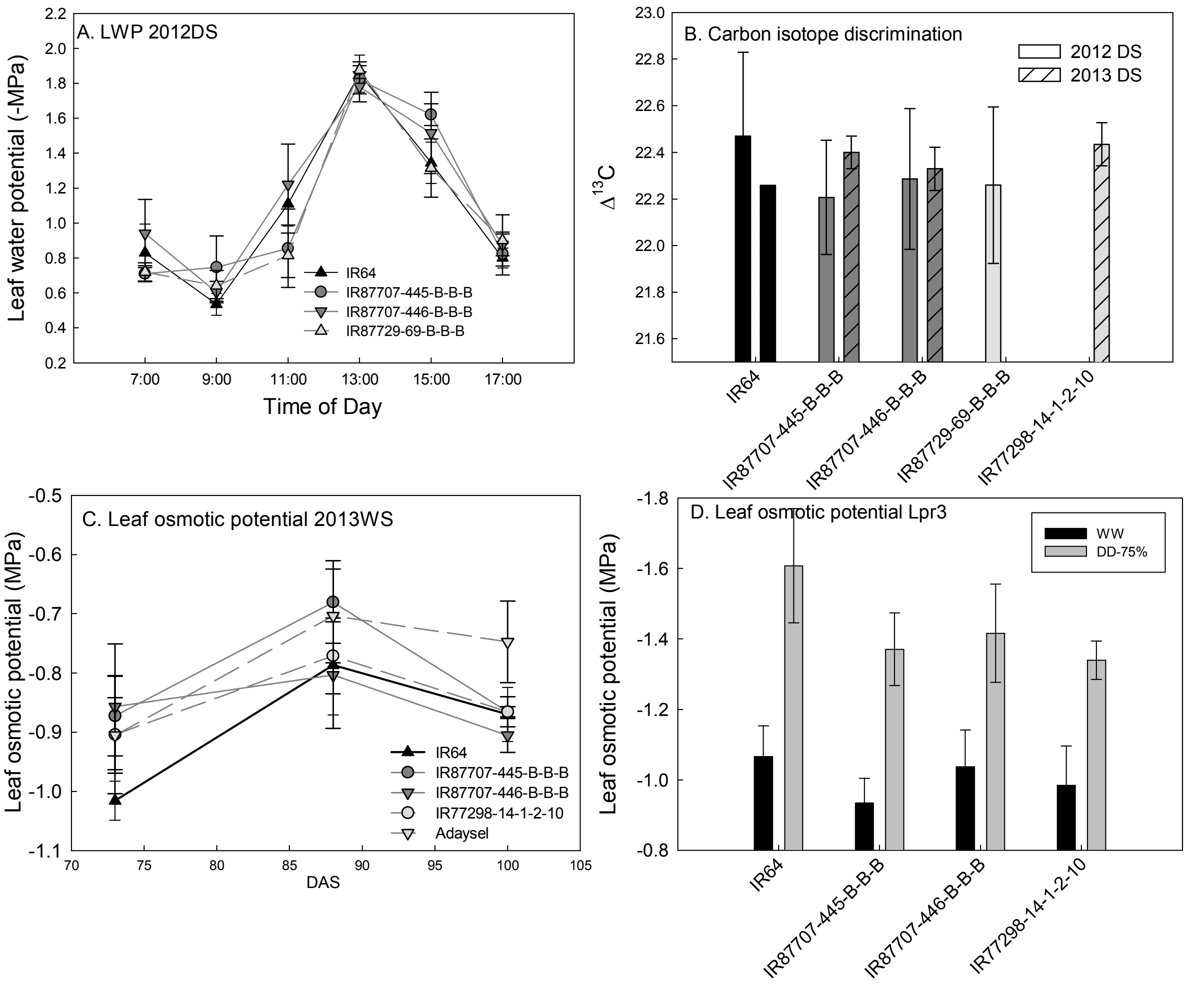

Supp. Fig. 8. Leaf water status in the WW treatment according to A) leaf water potential over the course of the day (88 DAS), B) carbon isotope discrimination (79 DAS in 2012DS, 114 DAS in 2013DS), and C) leaf osmotic potential. D) Leaf osmotic potential in the Lpr3 seedling stage greenhouse study. IR64 is indicated by a black line/bar, and the 2-QTL NILs are indicated by dark gray lines/bars in panels A-C.

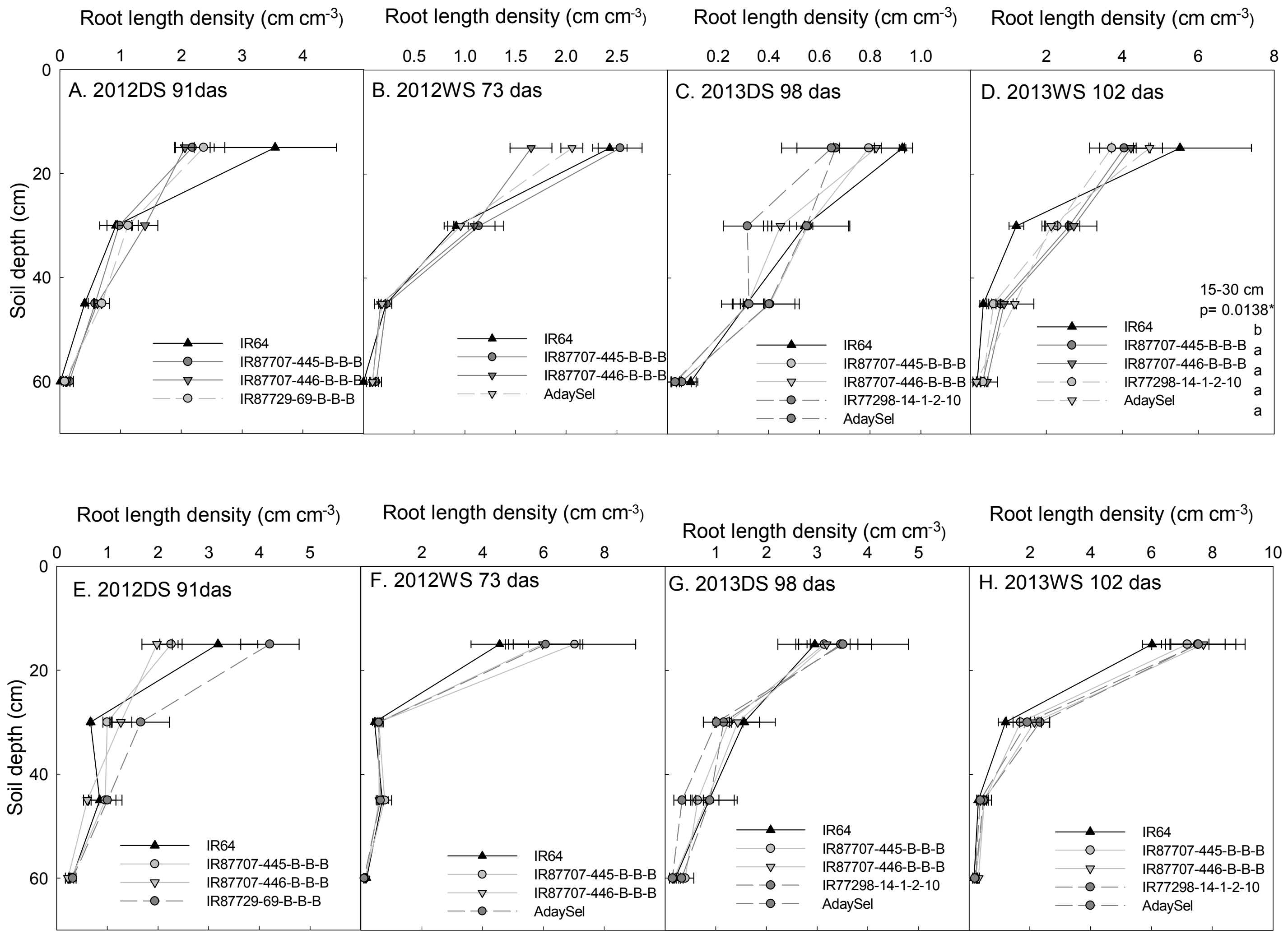

Supp. Fig. 9. Distribution of root length density with depth in the drought stress (A-D) and well-watered (E-H) treatments of the field studies. IR64 is indicated by black lines, and the 2-QTL NILs are indicated by dark gray lines.

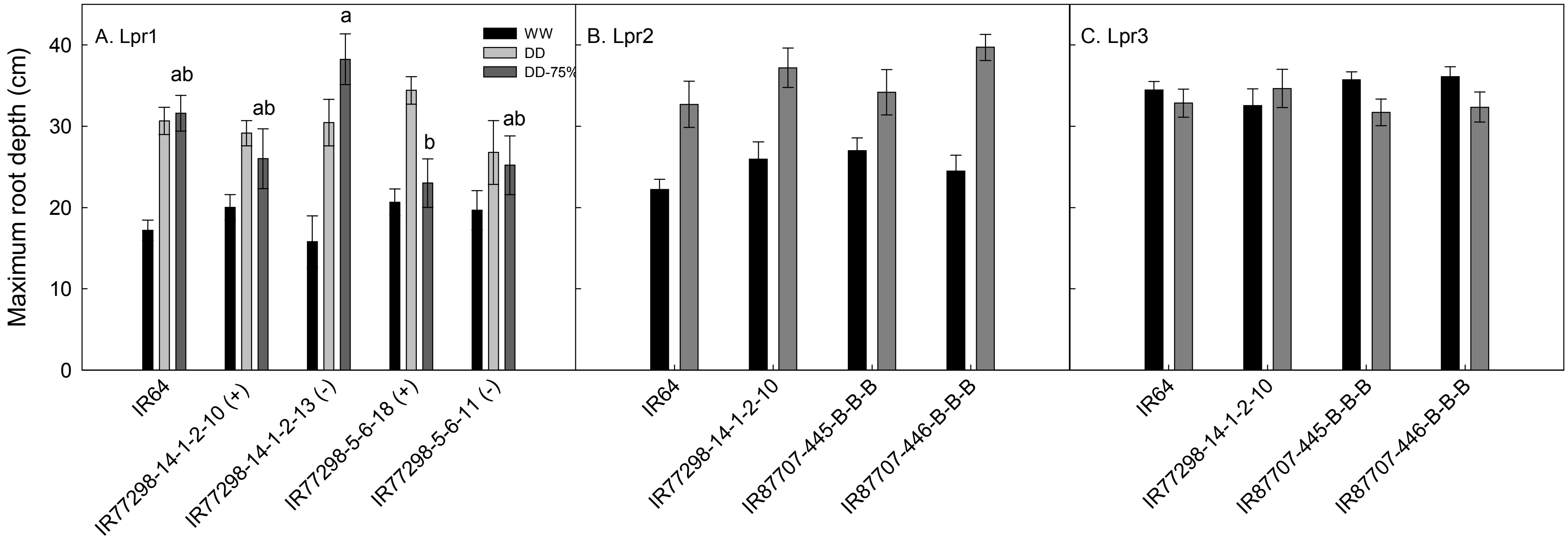

Supp. Fig. 10. Maximum root depth in greenhouse studies (A-C). Values shown are mean ± se, and letters indicate significant differences among genotypes as determined by LSD comparison (P<0.05).

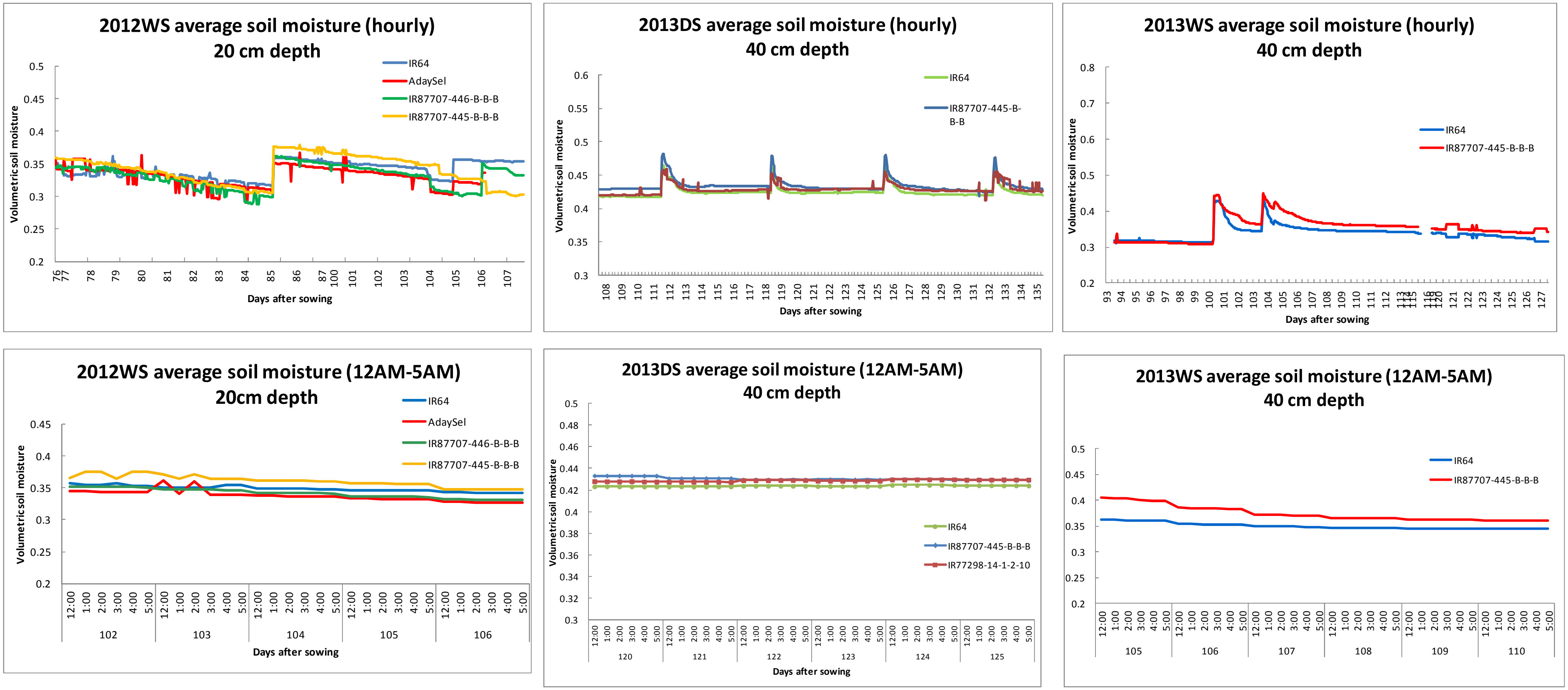

Supp. Fig. 11. Trends in soil moisture across the seasons 2012WS, 2013DS, and 2013WS (A, B, C) and diurnal trends (D, E, F) did not indicate genotypic differences in water uptake or that night-time water loss from roots to the soil was occurring.

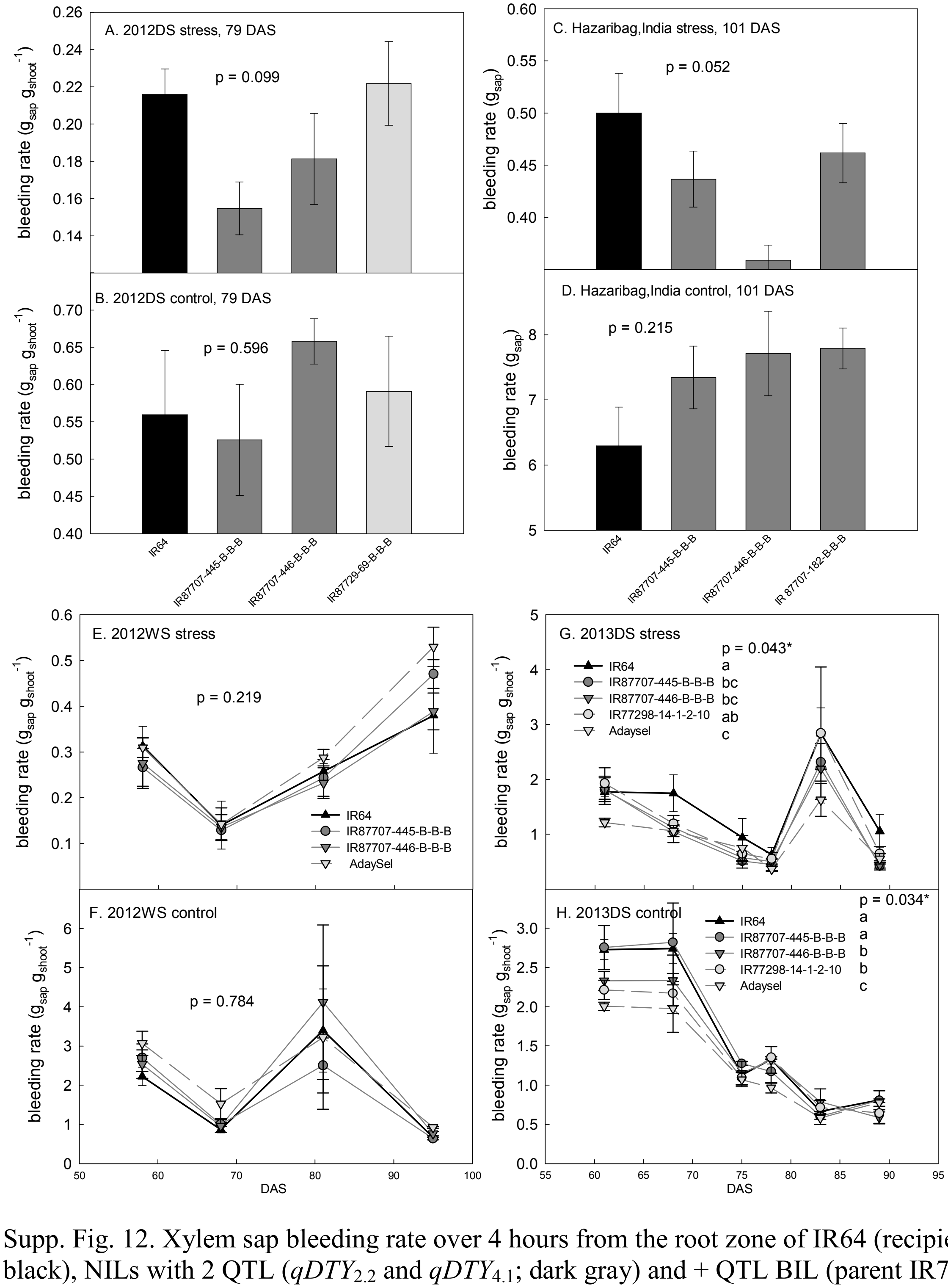

Supp. Fig. 12. Xylem sap bleeding rate over 4 hours from the root zone of IR64 (recipient parent; black), NILs with 2 QTL (*qDTY*$_{2.2}$ and *qDTY*$_{4.1}$; dark gray) and + QTL BIL (parent IR77298-14-1-2-10), +4QTL NIL IR87729-69-B-B-B, and donor AdaySel; light gray). Values shown are mean ± se, and letters indicate significant differences among genotypes as determined by LSD comparison (P<0.05). P-values shown are for genotypic differences across the experiment as determined by the mixed model ASREML. IR64 is indicated by a black line/bar, and the 2-QTL NILs are indicated by dark gray lines/bars.
